# Hepatic Circadian-Clock System Altered by Insulin Resistance, Diabetes and Insulin Sensitizer in Mice

**DOI:** 10.1371/journal.pone.0120380

**Published:** 2015-03-23

**Authors:** Huey-Ling Tseng, Shu-Chuan Yang, Shih-Hsien Yang, Kun-Ruey Shieh

**Affiliations:** 1 Institute of Medical Sciences, School of Medicine, Tzu Chi University, Hualien, Taiwan; 2 Department of Physiology, School of Medicine, Tzu Chi University, Hualien, Taiwan; 3 General Education Center, Tzu Chi College of Technology, Hualien, Taiwan; 4 Department of Rehabilitation Medicine, Buddhist Tzu Chi General Hospital, Hualien, Taiwan; University of Lübeck, GERMANY

## Abstract

Circadian rhythms are intrinsic rhythms that are coordinated with the rotation of the Earth and are also generated by a set of circadian-clock genes at the intracellular level. Growing evidence suggests a strong link between circadian rhythms and energy metabolism; however, the fundamental mechanisms remain unclear. In the present study, neonatal streptozotocin (STZ)-treated mice were used to model the molecular and physiological progress from insulin resistance to diabetes. Two-day-old male C57BL/6 mice received a single injection of STZ and were tested for non-obese, hyperglycemic and hyperinsulinemic conditions in the early stage, insulin resistance in the middle stage, and diabetes in the late stage. Gene expression levels of the hepatic circadian-clock system were examined by real-time quantitative PCR. Most of the components of the hepatic circadian-clock gene expression system, such as the mRNAs of *Bmal1* (brain and muscle Arnt-like protein-1), *Per2* (period 2) and *Cry1* (cryptochrome 1), were elevated, and circadian patterns were retained in the early and middle stages of insulin-resistant conditions. The insulin sensitizer, rosiglitazone, returns the physiological and molecular changes associated with the diabetic phenotype to normal levels through peroxisome proliferator-activated receptor γ (PPARγ) rather than PPARα. Early and chronic treatment with rosiglitazone has been shown to be effective to counter the diabetic condition. Over time, this effect acts to attenuate the increased gene expression levels of the hepatic circadian-clock system and delay the severity of diabetic conditions. Together, these results support an essential role for the hepatic circadian-clock system in the coordinated regulation and/or response of metabolic pathways.

## Introduction

The metabolic syndrome is characterized by a clustering of metabolic abnormalities, including central obesity, dyslipidemia, hypertension and systemic insulin resistance (IR), that together confers an increased risk of developing type 2 diabetes mellitus (DM) [1]. Although the metabolic syndrome is readily identified, its pathophysiological mechanisms are complex. IR seems to play a significant role in its pathogenesis [2] because DM subjects can be lean but have IR [3]. Using non-obese animal models, elucidating the mechanisms underlying the influence of IR or DM in the absence of obesity is one of the main aims of this study.

The liver aids the organism via temporally tuning expression of genes in relation to food supply. Metabolic regulation is a dynamic process within the hepatic system and is triggered by sensing nutrient stimuli and producing various nutrients. Liver-specific insulin receptor knockout mice exhibit intense IR, severe glucose intolerance and striking hyperinsulinemia [4]. In contrast, glucose homeostasis remains normal in mice with disrupted insulin signaling in both skeletal muscle and adipose tissue [5]. Thus, the liver is critical in regulating and maintaining glucose homeostasis, including gluconeogenesis, and the circadian-clock system is thought to be involved in these processes which coordinate internal glucose metabolism based on external stimuli [6–8].

Circadian rhythmicity exists in mammals and is essential in the response to numerous physiological requests [9, 10]. The circadian rhythm is driven by the molecular circadian-clock system, which includes *Bmal1* (brain and muscle aryl-hydrocarbon receptor nuclear translocator-like protein-1), *Clock* (circadian locomotor output cycles kaput), *Per1* (period 1), *Per2*, *Per3*, *Cry1* (cryptochrome 1) and *Cry2* [9, 10]. This system involves interacting positive and negative transcriptional/translational feedback loops that are composed of a set of circadian-clock genes that encode highly conserved transcription factors and enzymes that generate rhythmic expression [9, 10]. These positive and negative feedback loops of circadian-clock genes drive the circadian physiological output by regulating the expression of downstream circadian-clock-controlled genes, such as *Dbp* (albumin D-site-binding protein) and *E4BP4* (basic leucine zipper transcription factor). This circadian-clock system exists throughout the entire body, including the heart, lung, kidney and liver [11–13], although the physiological role of circadian-clock genes in peripheral tissues is not fully understood. However, the impact on skeletal muscle, pancreatic islets and adipose tissues with respect to the regulation of glucose metabolism is becoming clearer [14–16].

The peroxisome proliferator-activated receptor α (PPARα) and γ (PPARγ) are nuclear receptors expressed predominantly in the liver during both the fed and fasted state. PPARα and PPARγ agonists are applied as the anti-hyperlipidemic and anti-diabetic drugs, and are also involved in hepatic glucose metabolism. PPARα exerts a direct regulation on gluconeogenesis through stimulation of pyruvate dehydrogenase kinase 4 (PDK4) [17]. In contrast, treatment with PPARγ agonists decreases expression of gluconeogenesis related genes, and mice with the liver-specific disruption of *PPARγ* develop IR [18]. This indicates that the liver is also the major target of thiazolidinediones (TZDs), an insulin sensitizer and synthetic class of compounds that bind to PPARγ and are widely used to treat type 2 DM. Furthermore, hepatic phosphoenolpyruvate carboxykinase (PEPCK) and PDK4, which are involved in maintaining glucose homeostasis, are also regulated by the circadian-clock system [11, 19–21]. The PPARγ coactivator-1α (PGC-1α), which acts as an important integrator between the circadian-clock system and energy metabolism, is required for maintaining circadian glucose homeostasis during gluconeogenesis and the hepatic fasting response [22]. Disruptions of circadian-clock systems related to metabolic abnormalities have been reported. These findings showed that *Bmal1-*deficient or *Clock-*mutant mice exhibited impaired glucose metabolism and the metabolic syndrome [16, 23]. Pancreatic islet specific *Bmal1* knockout mice developed overt type 2 DM [15]. These studies provide potential therapeutic relevance linking the circadian-clock system and the metabolic syndrome. Treatment with the insulin sensitizer rosiglitazone and analysis of the pathophysiological progress and gene expression in neonatally streptozotocin (STZ)-treated male mice demonstrates the importance of the hepatic circadian-clock system in relation to glucose metabolism.

## Materials and Methods

### Animals

Timed pregnant C57BL/6 mice were purchased from the National Laboratory Animal Center (Taipei, Taiwan). Male neonatal offspring (two-day old) received an intraperitoneal administration of STZ (60 mg/kg body weight (BW); Sigma-Aldrich, St. Louis, MO, USA) that was freshly dissolved in 0.1 M citrate buffer at zeitgeber time 2 (ZT2), with ZT0 defined as lights-on [24]. The controls were injected with 0.1 M citrate buffer. The blood glucose of all neonates (three-day old) was measured by the ACCU-CHEK Blood Glucose Meter System (Roche Diagnostics, Basel, Switzerland). Only animals with blood glucose levels over 250 mg/dl were used in the protocol. Animals at two-weeks of age with a fasting (8 hours) blood glucose below 150 mg/dl and without glycosuria (test strips; Keto-Diastix, Bayer Diagnostics Manufacturing Ltd., South Wales, UK) were used in this study as the PND2-STZ group. Only 50% of the animals were enrolled in this study after being administered a single intraperitoneal injection of STZ as the PND2-STZ group.

The control and PND2-STZ groups at 30- or 46-weeks old received daily saline or 0.5 ml treatments at ZT11 by oral gavages for two weeks. The treatments included rosiglitazone (Rosi, 4 mg/kg; GalxoSmithKline, Burgos, Spain), Rosi with PPARγ antagonist GW9662 (4 mg/kg; Sigma-Aldrich), or Rosi with PPARα antagonist GW6471 (8 mg/kg; Sigma-Aldrich) in fresh saline. For the experiment with chronic Rosi treatment, Rosi was prepared fresh daily with water containing 0.1% dimethylsulfoxide (DMSO; Sigma-Aldrich) at a concentration of 0.2 mg/ml and used within 2 h after ZT12. The average drinking volume in 2 h post ZT12 treated animals was 1.0~2.1 ml, and the intake dosage was approximately 0.2~0.42 mg/per day in 20- to 60-week old animals. To eliminate the light cue, all animals were kept in constant darkness for 48 hours before tissue collection. Animals were euthanized with CO_2_ inhalation under dim red light (<1 lux) for tissue collection in constant darkness at ZT2, 6, 10, 14, 18, and 22. All procedures were approved by the Institutional Animal Care and Use Committee in Tzu Chi University. The institutional guidelines were followed for the care and use of animals.

### Blood chemistry and hormone measurements

Samples for fasting blood and insulin levels were obtained from the orbital sinus at ZT3 and measured by ACCU-CHEK and ultrasensitive mouse insulin enzyme-linked immunosorbent assay kits (Mercodia, Uppsala, Sweden). For the glucose tolerance test, blood glucose levels after overnight fasting were measured at 0, 30, 60, 120 and 180 min after administering 50% dextrose (2 g/kg BW). For the insulin tolerance test, blood glucose levels after overnight fasting were also measured at 0, 30, 60, 90 and 120 min after administering human regular insulin (0.25 U/kg BW, Actrapid; Novo Nordisk, Bagsvaerd, Denmark). The baseline (100%) was recorded at 0 min.

### RNA extraction and real-time quantitative PCR detection

Total RNA was extracted with the Trizol reagent (Gibco-BRL, Grand Island, NY, USA) and converted into complementary DNA by the ImProm-II Reverse Transcription System (Promega, Madison, WI, USA) with oligo(dT)_20_ and random hexamer primers according to the manufacturer’s instructions. Real-time quantitative PCR was performed in triplicate with a Chromo4 Continuous Fluorescence Detector (Bio-Rad, Hercules, CA, USA) and 2× Maxima SYBR Green/ROX qPCR Master Mix (Fermentas, Burlington, Ontario, Canada) and 0.2 μM primers (listed in S1 Table). To minimize the differences among different plates for the detection of the same gene, each plate shared a constant sample. Relative gene expression levels were determined using the comparative C_T_ method to normalize target genes to β-actin, as in previous studies [11, 21].

### Western blotting

Briefly, homogenization buffer was added to tissues which were then homogenized on ice, centrifuged, and the supernatant carefully removed. Basing on Bradford protein quantification, the 50 μg protein lysate was electrophoretically fractionated through a 12% SDS-polyacrylamide gel and electrotransferred onto a polyvinylidene difluoride (PVDF) membrane (Amersham, Piscataway, NJ, USA). The blots were soaked in blocking buffer for one hour at room temperature to block non-specific protein-binding sites. The primary antibodies for BMAL1 (EMD Millipore, Billerica, MA, USA; 1/2500), PPARγ (Thermo Fisher Scientific, Waltham, MA, USA; 1/4000) and β-Actin (EMD Millipore; 1/20000) were diluted in TBST buffer according to the titer of the antibodies. The horseradish peroxidase-conjugated secondary antibodies (EMD Millipore; 1/4000) were diluted in TBST. Detection of the immunoreactive components on the blots were performed by using the ECL (Santa Cruz, CA, USA) Western Blotting detection reagent depending on the sensitivity of the antibodies.

### Statistical analysis

All values are expressed as the means ± SEM. Differences in BW and the levels of fasting blood glucose and serum insulin were evaluated by two-way ANOVA with repeated measurement and followed by the post-hoc Newman-Keuls multiple comparison test. The trapezoidal rule was used to determine the area under curve (AUC) and the difference of AUC was evaluated by one-way ANOVA followed by the post-hoc Newman-Keuls multiple comparison test. Differences in relative gene and protein expression levels were evaluated by the two-way ANOVA followed by the post-hoc Newman-Keuls multiple comparison test. Circadian rhythmicity was determined by cross-sectional analysis using the Cosinor procedure that assumed a 24 h period as described previously [25]. The acrophase (time of peak expression), mesor (rhythm-adjusted mean), amplitude of rhythmicity, percentage of rhythm and significance of fit to a 24 h period (as indicated by the *p* value) for the expression levels of each gene were extrapolated from the freely available program Cosinor (http://www.circadian.org/softwar.html). A *p* value below 0.05 was considered significant.

## Results

### Changes in BW, fasting blood glucose, serum insulin, glucose tolerance and insulin tolerance in male PND2-STZ mice

To study the progress of IR and DM in neonatally STZ-treated male mice, the BW, blood glucose, insulin levels, and glucose and insulin tolerance were examined. The PND2-STZ group developed IR status between 20- to 40-weeks old and DM status between 44- to 48-weeks old. From two- to 43-weeks old, the BW was similar between the control group and the PND2-STZ group (S1A Fig.; *p*>0.05). The BW of the PND2-STZ group significantly decreased after 44-weeks old of age (S1A Fig.; *p*<0.05). The fasting blood glucose in the PND2-STZ group was higher than that of the control group (S1B Fig.; *p*<0.05), but not compared to those at 12- to 16-weeks old of age (S1B Fig.). Serum insulin levels fluctuated below the control levels at 4–8 and 44–48 weeks old of age. Serum insulin levels were higher at 20-~40-weeks old in the PND2-STZ group (S1C Fig.; *p*<0.05). There was no significant difference between groups in blood levels of both triglycerides and total cholesterol (data not shown). Only the animals in the PND2-STZ group showed glycosuria at 44- and 48-weeks old. The 20–40 week old PND2-STZ mice showed higher fasting blood glucose and serum insulin levels, but not glycosuria. The intraperitoneal glucose and insulin tolerance tests were used to verify whether the PND2-STZ group had developed IR after 16-weeks.

After overnight fasting, the blood glucose levels at all time points for both groups post 50% dextrose administration were similar at 16-weeks old (S2A Fig.; *p*>0.05). Blood glucose levels showed a significant elevation in the PND2-STZ mice at 24-weeks old (S2B–E Fig.; *p*<0.05). The blood glucose levels at 0 and 120 min in the PND2-STZ group were higher after 50% dextrose treatment at 24-weeks old (S2B Fig.; *p*<0.05). At 32-weeks old, the blood glucose levels in the PND2-STZ group were higher at 0, 30, 60 and 120 min, except at 180 min, following 50% dextrose injection (S2C Fig.; *p*<0.05). At 40- and 48-weeks old, the blood glucose levels of the PND2-STZ group were significantly higher at all time points following 50% dextrose administration (S2D–E Fig.; *p*<0.05). Using the intraperitoneal insulin tolerance test, the changes in blood glucose levels after insulin treatment were similar at 16-weeks old (S2F Fig.; *p*>0.05). Differences in blood glucose levels after insulin treatment were evident at 24-~32-weeks old (S2G–J Fig.; *p*<0.05). The ratios of the changes in blood glucose levels post insulin injection were greater at 60 min in the PND2-STZ group at 24-weeks old (S2G Fig.; *p*<0.05). At 32-weeks old post insulin injection the blood glucose levels were greater at 30 and 60 min than in the control (S2H Fig.; *p*<0.05). The ratios of the change in blood glucose levels at all time points in the PND2-STZ group at 40- and 48-weeks old post insulin injection were significantly higher (S2I–J Fig.; *p*<0.05). According to the above data (S1–S2 Figs.), the PND2-STZ group developed IR status between 20- to 40-weeks old. The DM status appeared approximately 44- to 48-weeks old, therefore, animals in the 32- and 48-week age range were chosen for the following experimental design. These animals are referred to as the IR group and rather that the PND2-STZ group at 32-weeks old. The PND2-STZ groups at 48-weeks old are referred to as the DM group. The IR and DM groups are used in the following figures and tables to differentiate the disease statuses.

### Rosiglitazone (Rosi) attenuated the IR status but had few effects on the DM status

**Fig 1:**
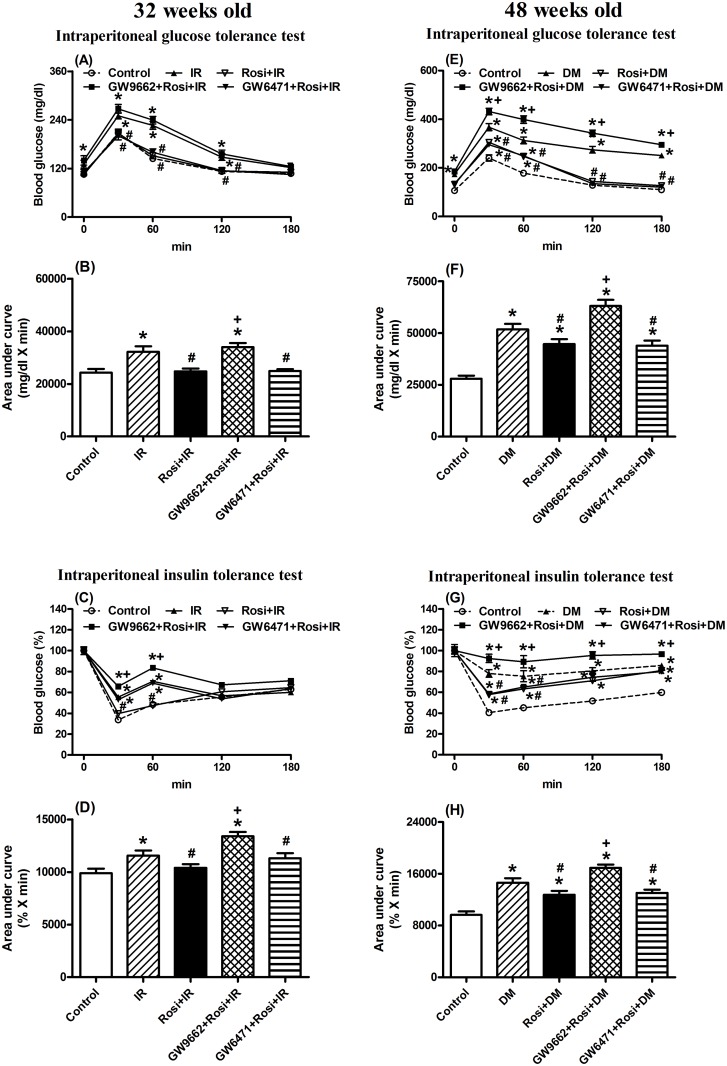
Effect of rosiglitazone (Rosi) treatment with either PPARα antagonist (GW6471) or PPARγ antagonist (GW9662) for two weeks on the blood glucose response to intraperitoneal glucose tolerance (A-B, E-F) and intraperitoneal insulin tolerance (C-D, G-H) on 32 weeks old mice (A-D) with insulin resistance (IR) or 48 week old mice (E-H) with diabetes (DM). Values are presented as the mean ± SEM (5~6 animals for each time point in A, C, E and G panels; 35 animals for the control group and 35~36 animals for the IR or DM groups with further treatments in B, D, F and H panels). * *p*<0.05 compared to mice injected with citrate buffer as the controls; ^#^ *p*<0.05 compared with the IR or DM groups; + *p*<0.05 compared to mice with Rosi treatments. Open circle: the control group; filled triangle: IR or DM; reversed open triangle: IR or DM treated for two weeks with Rosi; filled square: IR or DM treated for two weeks with Rosi and PPARγ antagonist, GW9662; reversed filled triangle: IR or DM treated for two weeks with Rosi and PPARα antagonist, GW6471.

To verify the specific effects of rosiglitazone (Rosi) on glucose and insulin tolerance, the PPARγ antagonist (GW9662) and PPARα antagonist (GW6471) were used during IR at 32-weeks old and DM at 48-weeks old. Rosi improved the IR status but had a minor effect on the DM status. The IR and control groups both received the same treatments. The control groups with treatments showed no significant differences in the intraperitoneal glucose tolerance tests (S3A–B Fig and S3E–F Fig.; *p*>0.05) or in the intraperitoneal insulin tolerance tests (S3C–D Fig and S3G–H Fig.; *p*>0.05). The IR group at 32-weeks old following the 50% dextrose treatment at time 0 to 120 min showed elevated blood glucose according to the intraperitoneal glucose tolerance test (Fig. 1A; *p*<0.05). After two weeks of Rosi treatment in the IR group, blood glucose levels were restored to the same levels as the controls (Fig. 1A; *p*>0.05). Animals treated with Rosi and PPARγ antagonist (GW9662) for two weeks showed elevated blood glucose levels compared to the control group (Fig. 1A; *p*<0.05). The results of the co-treatments of PPARα antagonist (GW6471) with Rosi for two weeks were similar to those in the IR group treated with Rosi only and the controls (Fig. 1A; *p*>0.05).

This is also illustrated by the AUC measurement (Fig. 1B; *p*<0.05). The AUC_glucose_ in the IR group at 32-weeks old was higher (Fig. 1B; *p*<0.05), whereas IR animals treated with Rosi were restored to the same levels as the controls (Fig. 1B; *p*>0.05) and were lower than that of the IR only group (Fig. 1B; *p*<0.05). The AUC_glucose_ in the IR group with co-treatment of GW9662 and Rosi was higher than those in the control and IR only groups (Fig. 1B; *p*<0.05). The IR group treated with Rosi was restored to the same levels as the controls (Fig. 1B; *p*>0.05) and was lower than that the IR only group (Fig. 1B; *p*<0.05). After co-treatment with Rosi and GW6471 for two weeks, the AUC_glucose_ was similar to that of the control group and the IR with Rosi only group (Fig. 1B; *p*>0.05). The AUC_glucose_ was lower than that in IR only group (Fig. 1B; *p*<0.05). In the intraperitoneal insulin tolerance test, the blood glucose levels after insulin treatment in the IR only group were higher at 32-weeks old (Fig. 1C; *p*<0.05). Rosi treatment improved the elevation of blood glucose levels following insulin treatment (Fig. 1C; *p*<0.05), and co-treatment with Rosi and GW9662 reversed the improvement of Rosi only treatment (Fig. 1C; *p*<0.05). In contrast, the co-treatment with Rosi and GW6471 had no effect on the Rosi effect and showed similar responses as the Rosi only group (Fig. 1C; *p*>0.05). The results of the AUC_glucose_ analysis (Fig. 1D) were similar to the ratios of blood glucose levels (Fig. 1C), and co-treatment with Rosi and GW9662 reversed the improvement of Rosi only treatment (Fig. 1D; *p*<0.05).

The 48-week old male mice in the IR group developed DM status and exhibited: 1) decreased BW, 2) higher fasting glucose levels, 3) lower serum insulin levels, 4) glucose intolerance and 5) glycosuria (S1–S2 Figs.). These animals were used as the DM group instead of the IR group at 48-weeks old. In the intraperitoneal glucose test, the blood glucose levels in the Rosi treated group was slightly lower than those in the DM group, yet remained higher than those in the control group over time (Fig. 1E-F; *p*<0.05). The results of co-treatment with GW6471 and Rosi were similar to those in the Rosi only group (Fig. 1E-F), but co-treatment with GW9662 and Rosi blocked the Rosi effect and showed a greater elevation than the Rosi only group (Fig. 1E-F; *p*<0.05). Similar responses were also shown in the intraperitoneal insulin test (Fig. 1G-H).

### Rosi attenuated the gene expression changes of the hepatic circadian-clock and other genes in the IR status, but had few effects on those in the DM status

**Fig 2:**
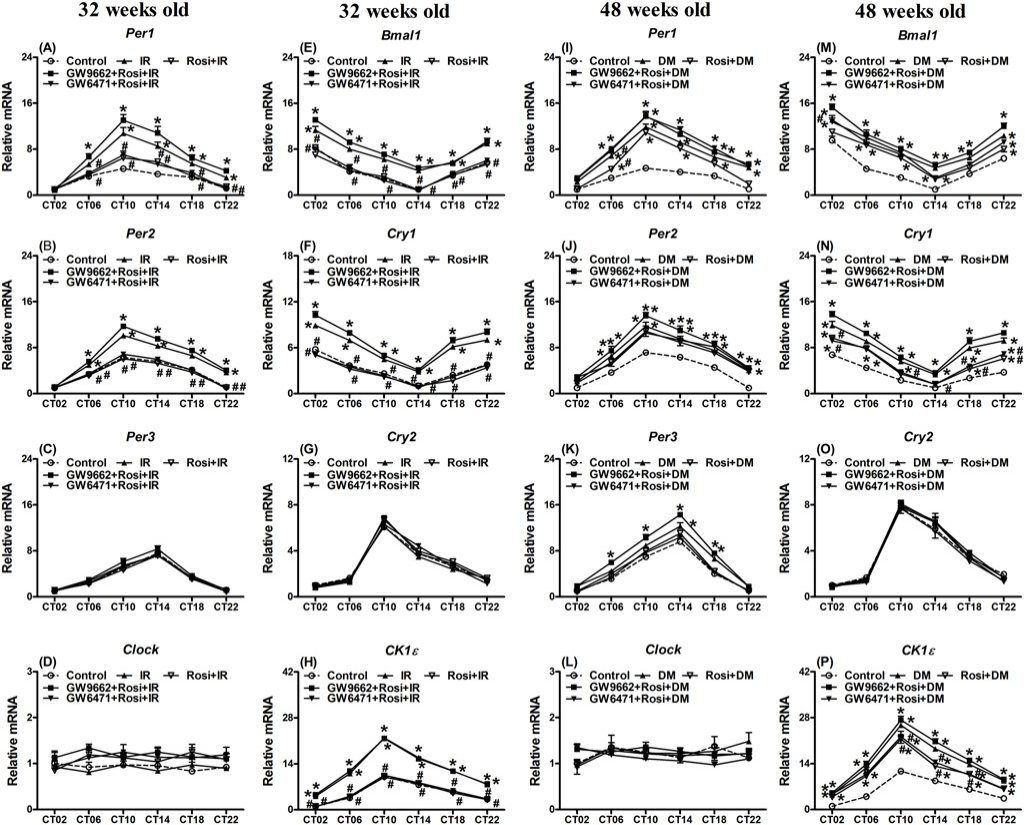
Expression levels of (A,I) *period 1* (*Per1*), (B,J) *Per2*, (C,K) *Per3*, (D,L) *circadian locomotor output cycles kaput* (*Clock*), (E,M) *brain and muscle Arnt-like protein-1* (*Bmal1*), (F,N) *cryptochrome 1* (*Cry1*), (G,O) *Cry2* and (H,P) *casein kinase 1ε* (*CK1ε*) in the livers of 32 weeks old mice with insulin resistance (IR) undergoing rosiglitazone (Rosi) treatment (left panel) or mice at 48 weeks old with diabetes (DM) undergoing Rosi treatments (right panel). Values are presented as the mean ± SEM (6 animals for each time point). * *p*<0.05 compared to mice injected with citrate buffer as the controls at the same time points. ^#^ *p*<0.05 compared with the IR or DM group at the same time points. Open circle: the control group; filled triangle: IR or DM; reversed open triangle: IR or DM treated for two weeks with Rosi; filled square: IR or DM treated for two weeks with Rosi and PPARγ antagonist, GW9662; reversed filled triangle: IR or DM treated for two weeks with Rosi and PPARα antagonist, GW6471.

**Fig 3:**
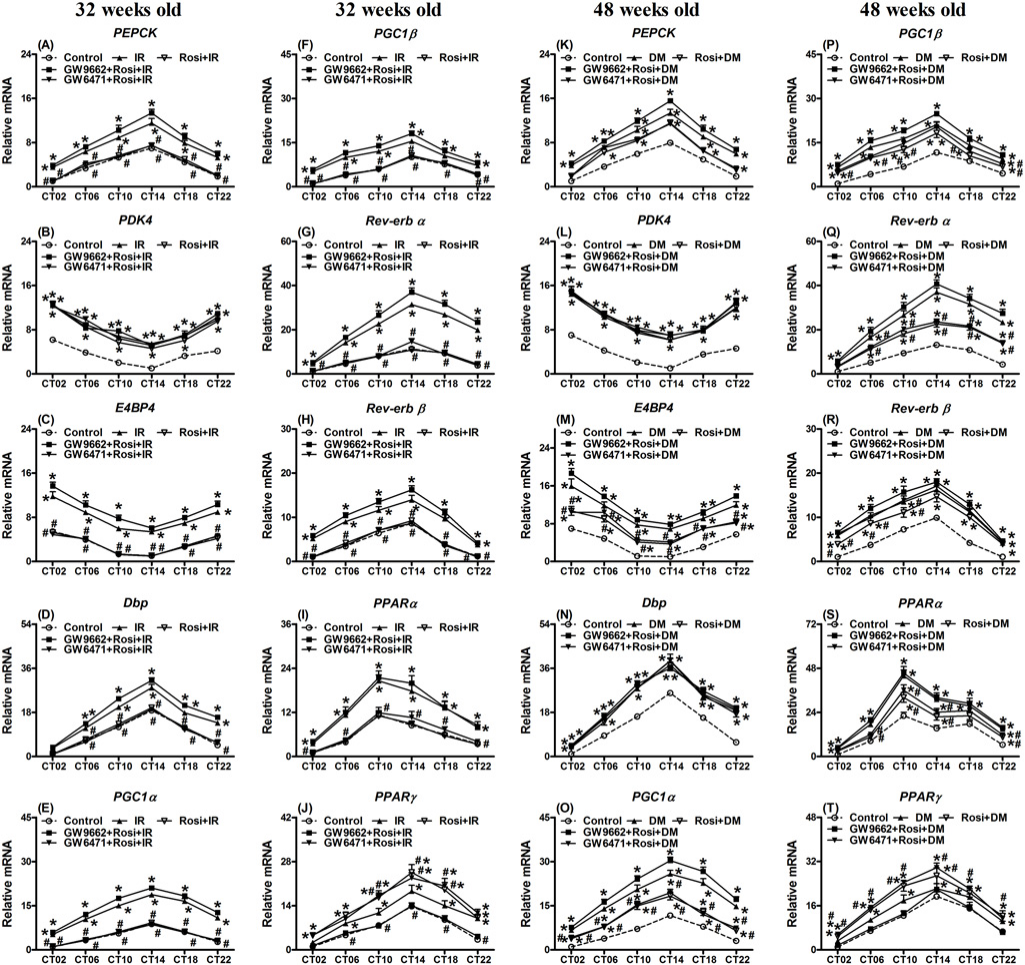
Expression levels of (A,K) *phosphoenolpyruvate carboxykinase* (*PEPCK*), (B,L) *pyruvate dehydrogenase kinase 4* (*PDK4*), (C,M) *basic leucine zipper transcription factor* (*E4BP4*), (D,N) *albumin D-site-binding protein* (*Dbp*), (E,O) *peroxisome proliferator-activated receptor-γ coactivator 1α* (*PGC1α*), (F,P) *1β* (*PGC1β*), (G,Q) *the transcription factor reverse erythroblastosis virus α* (*Rev-erb α*) and (H,R) *β* (*Rev-erb β*) as well as (I,S) *peroxisome proliferator-activated receptor α* (*PPARα*) and (J,T) *γ* (*PPARγ*) in the livers of 32 weeks old mice with insulin resistance (IR) undergoing rosiglitazone (Rosi) treatment (left panel) or mice at 48 weeks old with diabetes (DM) undergoing Rosi treatment (right panel). Values are presented as the mean ± SEM (6 animals for each time point). * *p*<0.05 compared to mice injected with citrate buffer as the controls at the same time points. ^#^ *p*<0.05 compared with the IR or DM group at the same time points. Open circle: the control group; filled triangle: IR or DM; reversed open triangle: IR or DM treated for two weeks with Rosi; filled square: IR or DM treated for two weeks with Rosi and PPARγ antagonist, GW9662; reversed filled triangle: IR or DM treated for two weeks with Rosi and PPARα antagonist, GW6471.

To study the effects of Rosi, IR and DM statuses on the hepatic circadian-clock systems, hepatic gene expression was detected in the IR status at 32-weeks old and the DM status at 48-weeks old. Rosi was able to influence the hepatic circadian-clock and related gene expressions on both the IR and DM statuses. Most of the measured circadian-clock and energy metabolism-related gene expression in the liver exhibited circadian patterns at 32-weeks old in the IR status and at 48-week old in the DM status (Figs. 2–3).

The exception was *Clock* mRNA (Fig. 2D, L). Cosinor analysis also confirmed that the gene expression revealed a 24 h periodicity of rhythmicity (S2 Table; Figs. 2–3; *p*<0.05). Similar to the results of intraperitoneal glucose and insulin tolerance tests found in S3 Fig., the expression patterns of *Per1*, *Per2*, *Bmal1*, casein kinase 1ε (*CK1ε*) and *Cry1* mRNAs showed no difference irrespective of the treatment for both the 32- and 48-weeks old (S4 Fig.; *p*>0.05). The following results in Figs. 2–3 show the control data, IR, IR with Rosi alone, IR with Rosi and GW9662 and IR with Rosi and GW6471 (Figs. 2–3). The levels of gene expression in the liver at 32-weeks old affected by IR, and the response after treatments over two weeks were divided into three phases. 1) The IR status elevated levels of gene expression of *Per1*, *Per2*, *Bmal1*, *Cry1*, *CK1ε*, *PEPCK*, *E4BP4*, *Dbp*, *PGC1α*, *PGC1β*, the transcription factor reverse erythroblastosis virus α (*Rev-erb α*), *Rev-erb β* and *PPARα* mRNAs and Rosi reversed this elevation to the same levels as the control group. 2) Rosi showed no effect on the elevation of *PDK4* and *PPARγ* mRNAs by IR. 3) *Per3*, *Clock*, and *Cry2* mRNAs remained unaffected by the IR status and Rosi also had no effect. Co-treatment with the PPARγ antagonist (GW9662) and Rosi blocked the improved effect of Rosi on the *Per1*, *Per2*, *Bmal1*, *Cry1*, *CK1ε*, *PEPCK*, *E4BP4*, *Dbp*, *PGC1α*, *PGC1β*, *Rev-erb α*, *Rev-erb β*, *PPARα*, and *PPARγ* gene expression in the liver in 32-week old IR animals. Notably co-treatment with PPAR*α* antagonist (GW6471) and Rosi neither improved nor exacerbated the effects of Rosi on elevated gene expression levels (Figs. 2–3). The changes in the mesor and amplitude of gene expression in the IR group were resolved after Rosi treatment and the levels were restored to that of the controls (S2 Table).

The DM status at 48-weeks old showed similar elevations of gene expression as those in the IR group at 32-weeks old. However, the effects of Rosi treatment were variable (Figs. 2–3): 1) The Rosi effect on gene expression was nonexistent or decreased in the DM status. The elevations of *Per2*, *Per3*, *Clock*, *Cry2*, *PDK4*, *E4BP4*, *Dbp*, *PGC1α*, *PGC1β*, *Rev-erb α*, *PPARα*, and *PPAR*γ gene expression in the liver were unchanged post Rosi treatment (Figs. 2–3). The elevations of *Per1*, *Bmal1*, *Cry1*, *CK1ε*, *PEPCK* and *Rev-erb β* gene expression were attenuated by two weeks of Rosi treatments. Most of the gene expression levels in the DM status remained higher than those in the control group. 2) Due to the few beneficial effects of Rosi on gene expression, the results of co-treatment with the PPARγ antagonist (GW9662) or the PPAR*α* antagonist (GW6471) and Rosi were similar to the Rosi only treated group. The changes in the mesor and amplitude of the gene expression (S2 Table) were also similar to those shown in the Figs. 2–3.

**Fig 4:**
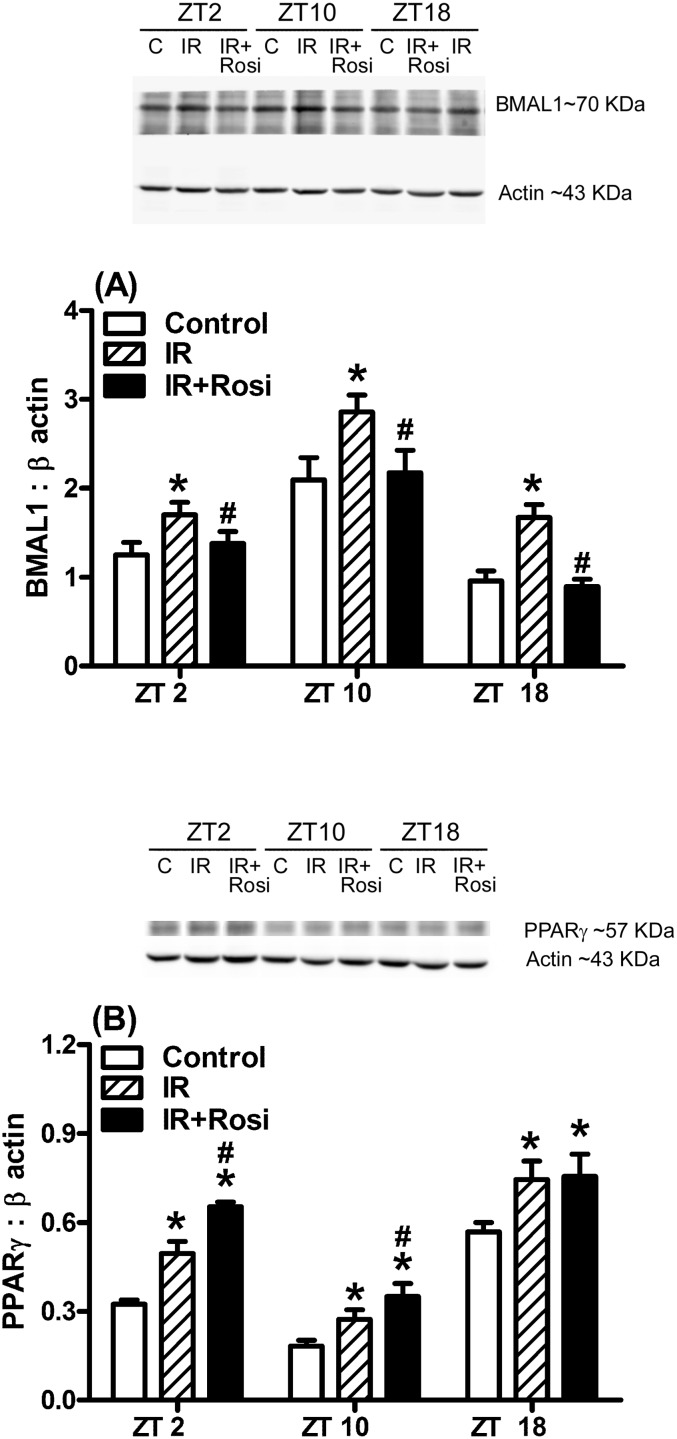
Levels of brain and muscle Arnt-like protein-1 (BMAL1) (A) and peroxisome proliferator-activated receptor γ (PPARγ) (B) protein in the livers of 32 week old mice with insulin resistance (IR) undergoing rosiglitazone (Rosi) treatment for two weeks. Values represent the mean ± SEM (3 animals for each group including the control (open bar), IR (striped) and IR + Rosi (filled)). * *p*<0.05 compared to mice injected with citrate buffer as the controls at the same time points. ^#^ *p*<0.05 compared with the IR group at the same time points.

To further verify whether the changes in protein levels were similar to the changes in gene expression, the BMAL1 and PPARγ protein levels in the livers were detected (Fig. 4).

BMAL1 and PPARγ protein levels exhibited the circadian patterns at 32-weeks old in the control, IR and IR with two-week treatment of Rosi conditions (Fig. 4; *p*<0.05). The highest expression of BMAL1 protein levels was found at ZT10 and the lowest was found at ZT18 (Fig. 4A; *p*<0.05). The BMAL1 protein levels in the control group at ZT2, ZT10 and ZT18 were elevated by IR status at 32-weeks old (Fig. 4A; *p*<0.05). Two-week treatment with Rosi attenuated the elevation of BMAL1 protein levels back to the levels of the control group (Fig. 4A; *p*<0.05). In addition, the highest expression of PPARγ protein levels in controls was found at ZT18 and the lowest was found at ZT2 (Fig. 4B; *p*<0.05). The PPARγ protein levels at ZT2, ZT10 and ZT18 were elevated by IR status at 32-weeks old (Fig. 4B; *p*<0.05). However, two-week treatment with Rosi did not attenuate the elevation of PPARγ protein levels back to the levels of control group, but showed greater elevation at ZT2 and ZT10 (Fig. 4B; *p*<0.05). These changes in protein levels (Fig. 4) were similar to the changes in mRNA expression (Figs. 2E and 3J).

### Chronic treatments with Rosi attenuated and/or delayed the IR status including the indices of blood chemistry and changes in the gene expression of the hepatic circadian-clock and other genes

**Fig 5:**
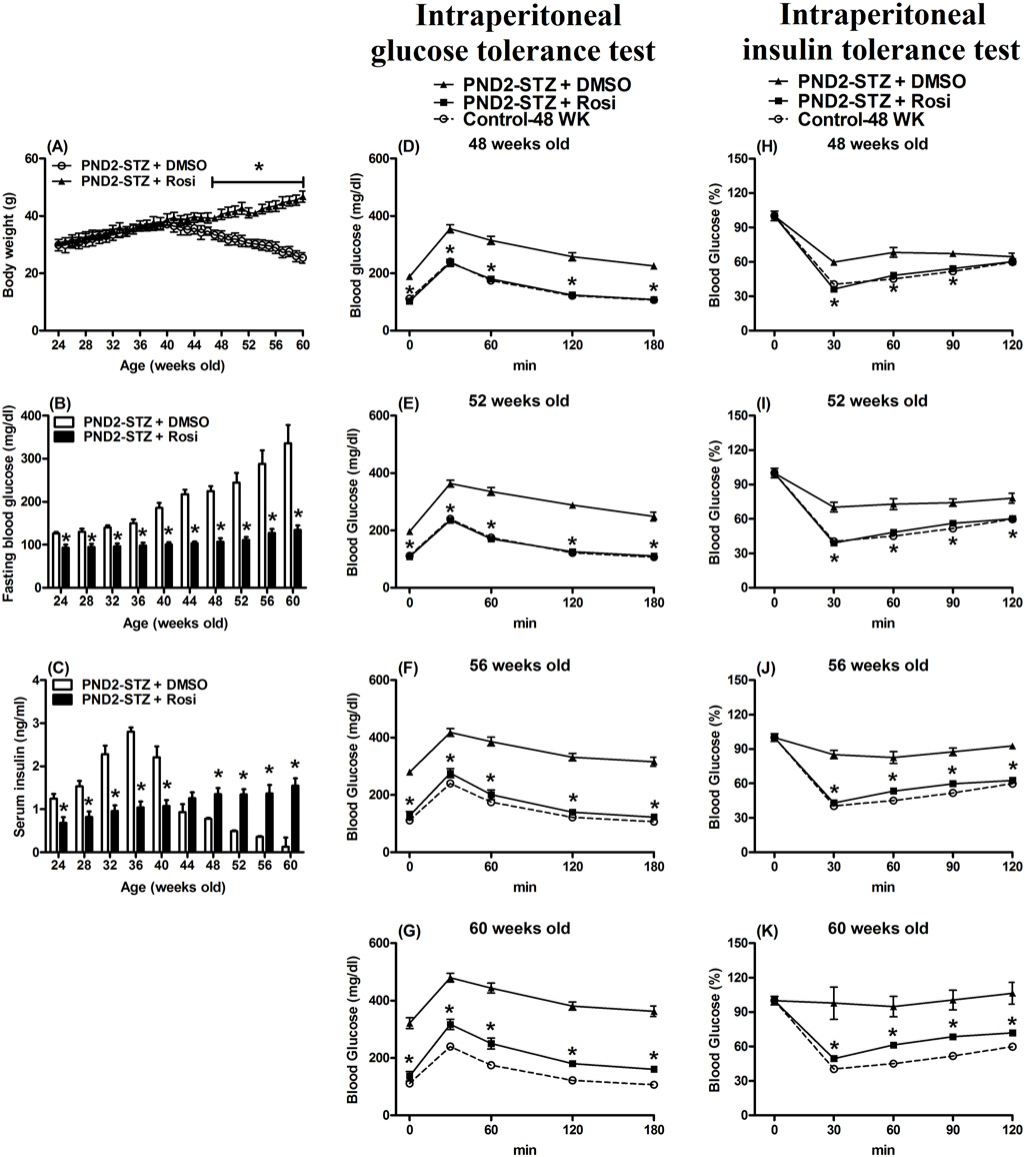
Effect of chronic rosiglitazone (Rosi) or vehicle (DMSO) treatments from 20 to 60 weeks of age on body weight (A), fasting blood glucose (B) and serum insulin (C) levels in postnatal day two-streptozotocin (PND2-STZ) treated male mice. The blood glucose response to intraperitoneal glucose tolerance (D-G) and intraperitoneal insulin tolerance tests (H-K) in mice at 48 (D,H), 52 (E,I), 56 (F,J) and 60 (G,K) weeks of age with postnatal day 2-streptozotocin (PND2-STZ) treatment undergoing chronic rosiglitazone (Rosi) or vehicle (DMSO) treatment from 20 to 60 weeks old. Values represent the mean ± SEM ((A) 82 animals for the PND2-STZ + DMSO group (open symbol); 93 animals for the PND2-STZ + Rosi group (filled)). * *p*<0.05 compared with mice that drank DMSO (PND2-STZ + DMSO) on the same day. ((D-K) 15~16 animals for each time point in the PND2-STZ + DMSO group (filled triangle); 17~19 animals for each time point in the PND2-STZ + Rosi group (filled square)). The open circle indicated the control group at 48 weeks old from the results in Fig. 1. * *p*<0.05 compared with mice that drank the DMSO (PND2-STZ + DMSO) at the same time points on the same day.

Due to the beneficial effects of two week (short-term) Rosi treatments, a long-term study was implemented. The long-term and early treatment of Rosi showed the better improvement in BW, blood glucose, insulin levels, and glucose and insulin tolerance tests as well as the hepatic circadian-clock and related gene expression on the IR and DM statuses. Twenty-week old PND2-STZ animals received Rosi in drinking water for 40 weeks. At 24-weeks old to 46-weeks old, the BW remained similar (Fig. 5A; *p*>0.05).

However, the BW of the PND2-STZ with DMSO group significantly decreased compared to that of the PND2-STZ with Rosi group after 47-weeks of age (Fig. 5A; *p*<0.05). All levels of fasting blood glucose in the PND2-STZ with Rosi group during the 24-~60-weeks period were lower (Fig. 5B; *p*<0.05). Serum insulin levels between the two groups were divided into two parts. From 24- to 40-weeks old, serum insulin levels in the PND2-STZ with Rosi group were lower (Fig. 5C; *p*<0.05), but from 48- to 60-weeks old they were higher than those in the PND2-STZ with DMSO group (Fig. 5C; *p*<0.05). The blood levels of both triglycerides and total cholesterol showed no difference between the two groups (data not shown), and only the animals in the PND2-STZ with DMSO showed glycosuria after 48 weeks of age.

After overnight fasting, the blood glucose levels of the 48-week old PND2-STZ with DMSO group post 50% dextrose administration were elevated (Fig. 5D-G; *p*<0.05). In the intraperitoneal insulin tolerance test, the ratios of changes in blood glucose level over time after insulin treatment in the PND2-STZ (52-weeks old) with Rosi group were also significantly lower (Fig. 5H-K; *p*<0.05). At 48-weeks old, the ratios of the blood glucose level were lower at 30, 60 and 90 min (Fig. 5H; *p*<0.05), but not at 120 min (Fig. 5H; *p*>0.05), in the PND2-STZ with Rosi group after insulin administration.

**Fig 6:**
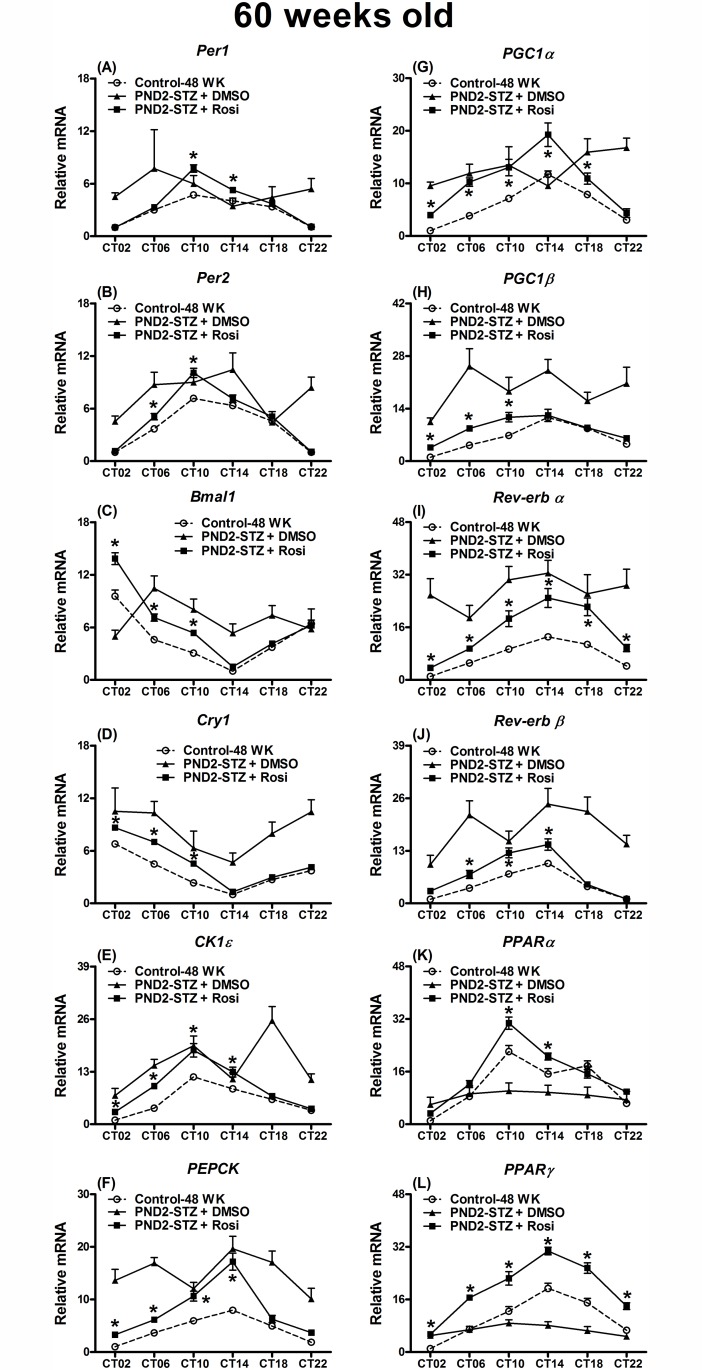
Expression levels of (A) *period 1* (*Per1*), (B) *Per2*, (C) *brain and muscle Arnt-like protein-1* (*Bmal1*), (D) *cryptochrome 1* (*Cry1*), (E) *casein kinase 1ε* (*CK1ε*), (F) *phosphoenolpyruvate carboxykinase* (*PEPCK*), (G) *peroxisome proliferator-activated receptor-γ coactivator 1α* (*PGC1α*), (H) *1β* (*PGC1β*), (I) *the transcription factor reverse erythroblastosis virus α* (*Rev-erb α*) and (J) *β* (*Rev-erb β*) as well as (K) *peroxisome proliferator-activated receptor α* (PPAR*α*) and (L) *γ* (PPARγ) in the livers of mice at 60 weeks old undergoing chronic rosiglitazone (Rosi) or vehicle (DMSO) treatment from 20 to 60 weeks old. Values are presented as the mean ± SEM (8~10 animals for each time point). Open circle: the control group at 48 weeks old from the results in Figs. 2–3; filled triangle: the PND2-STZ + DMSO group; filled square: the PND2-STZ + Rosi group. * *p*<0.05 compared to control mice injected with citrate buffer at 48 weeks old from the results in Figs. 2–3 at the same time points.

The levels of the circadian-clock and energy metabolism related gene expression in the liver at 60-weeks old were measured (Fig. 6).

Cosinor analysis was also used to verify whether the gene expression revealed a 24 h periodicity of rhythmicity (S2 Table). To reduce the animal numbers in this chronic experiment, we used the data from the control group at 48-weeks old in the previous experiment (Figs. 2–3) as the control (Fig. 6). In the PND2-STZ with DMSO group at 60-weeks old the loss of the 24 h levels of gene expression periodicity and rhythmicity was pronounced (S2 Table). These changes were noted as elevations at most time points compared to those in the PND2-STZ with Rosi or the control group at 48-weeks old (Fig. 6). The levels of gene expression in the PND2-STZ with Rosi group revealed a 24 h periodicity of rhythmicity and the mesor and amplitude were similar to the control group data at 48-weeks old (S2 Table). *Per1*, *Per2* and *PPARα* mRNA expression was elevated at ZT10~ZT14 or ZT6~ZT10. *Bmal1*, *Cry1* and *PGC1β* mRNAs were elevated at the periods from ZT2 to ZT10 compared to the data of the 48-week old control group (Fig. 6; *p*<0.05). *CK1ε*, *PEPCK*, *PGC1α* and *Rev-erb β* mRNA expression was elevated at the periods from ZT2 to ZT14, and *PPARγ* and *Rev-erb α* were elevated at all time points compared to the 48-week old control group (Fig. 6; *p*<0.05).

## Discussion

Growing evidence from human and animal studies has indicated that the circadian-clock system is linked to the regulation of energy metabolism. Many energy metabolism-related hormones, including insulin, glucagon, glucocorticoids and leptin exhibit circadian rhythmicity [26–29]. Recent evidence also suggests that alterations or imbalances in the settings of the circadian-clock system are possible causes of metabolic disorders [9]. Metabolic disturbances such as IR and DM are induced by irregularities of the circadian-clock system [9, 30]. This was noted in *Bmal1* deficient mice exhibiting impaired glucose and hepatic carbohydrate metabolism [16, 31, 32] as well as in *Clock-*mutant mice exhibiting obesity and metabolic syndrome [23]. In the study of tissue-specific ablation of *Bmal1*, it was demonstrated that the hepatic circadian-clock systems does indeed contribute to glucose homeostasis [14]. Alternatively, obesity, IR, and even DM also affect the balance of circadian-clock systems. High-fat diet treatments in male mice for six weeks [33] and 11 months [11] induced obesity and subsequent changes in hepatic circadian-clock gene expression. Changes in *Per2*, *Bmal1* and *Cry1* mRNA expression are related to human metabolic syndrome [34, 35]. A recent study showed the gene expression levels of the hepatic circadian-clock system are affected by IR alone without obesity [21]. Although the relationship between the circadian-clock system and energy balance is still unclear, the following mechanism has been suggested: Cellular energy levels such as the NAD^+^/NADH ratio may serve as links between energy metabolism and the circadian-clock system. This may occur by an interaction between the circadian-clock system via CLOCK-BMAL1 and the E-box [27, 36]. PPARγ is a key transcription factor involved in energy metabolism [37]. The PPARγ agonist Rosi is used for clinical therapy to treat IR and/or DM. The potential effect of Rosi on the hepatic circadian-clock system during the status of IR and/or DM was the main focus of this study.

STZ treatment in juvenile or adult animals is commonly used as a model for type 1 DM to study the circadian-clock system [38–40]. For example, a recent study showed that a decrease in locomotor activity at 12 weeks after the onset of DM in STZ-treated mice [41]. The incident rate of type 1 DM is lower than type 2 DM within the human population. Chronic high fat diet-induced type 2 DM is the most common initiating factor in human patients. Due to high cost and complications related to obesity in animal studies using high fat diet-induced DM, neonatal STZ treated mice were used to overcome these issues. The damage by STZ on the pancreatic β-cell is irreversible. However, pancreatic β-cell regeneration occurs in STZ treated neonates (before seven-days old) and these animals show glucose intolerance when they are adult due to the pancreatic β-cell numbers being reduced by 40% [42, 43]. In this study, two-day old male mice were treated with STZ and consequently developed IR and DM from 20-weeks old. During the period from 20- to 40-weeks old, these neonatal STZ-treated male mice showed higher fasting blood glucose and serum insulin levels as well as glucose and insulin intolerance. However, they exhibited the status of IR alone as the animals did not show glycosuria. These animals developed the status of DM after 44~48-weeks old and also exhibited higher fasting blood glucose, but lower serum insulin levels, and severe glucose and insulin intolerance as well as glycosuria. Compared to the model of chronic high fat diet-induced type 2 DM, these neonatal STZ-treated mice did not exhibit obesity or complications from adipokines or inflammation [44, 45]. As such, these neonatal STZ-treated male mice were appropriate to investigate the role of the hepatic circadian-clock system in the IR to DM progression without obesity-related complications. Based on the life span of these mice, the chronological similarity to humans and rates of IR and DM, the effect on the hepatic circadian-clock system may be further understood.

PPARγ is a nuclear receptor that binds to PPAR response elements in the regulatory region of target genes and involves various aspects of metabolism. PPARγ is a key regulator of glucose metabolism through improvement of insulin sensitivity. PPARγ activation is a therapeutic application that controls hyperglycemia and attenuates IR in type 2 DM. At the same time, disruption of PPARγ severely affects circadian rhythmicity. For example, systemic deletion of PPARγ in mice strongly altered the rhythmic patterns of food intake [46]. The expression of hepatic circadian-clock genes including *Bmal1*, *Clock*, *Per1*, *Per2*, *Per3*, *Cry1*, *Cry2*, and *Rev-erb α* were also affected by systemic deletion of PPARγ [46]. Importantly, changes in the circadian-clock gene expression in mice with systemic deletion of PPARγ were found only in the liver and not in the kidney or skeletal muscles [46]. The expression of *Bmal1*, *Per1*, *Cry1*, and *Rev-erb α* genes were affected by the statuses of IR and DM. Treatment with Rosi (PPARγ agonist) was able to attenuate or even reverse these effects. CRY1 regulates the activity of the CREB protein [8] which inhibits hepatic PPARγ expression [47] and/or enhances hepatic PGC-1α expression [48]. This implies that PPARγ interacts with the circadian-clock system to regulate energy metabolism. The beneficial effects of PPARγ agonists on IR and DM may be through the influence on the circadian-clock systems. Yang and his colleagues found that Rosi treatment for six weeks activates *PPARγ* mRNA expression [49]. Recent studies show that a high fat diet induces the reprogramming of the hepatic circadian-clock system partly through PPARγ [50]. These studies [49, 50] and the current study suggest that the hepatic circadian-clock system is mediated by PPARγ activation to regulate glucose balance. However, some differences between these studies [49, 50] and Hofmann et al. [40] were found, possibly due to the animal models used. The rhythmicity of *PPARγ* mRNA expression is impaired and suppressed in the type 1 DM model [40] but is enhanced or activated in obese or IR conditions.

In addition to PPARγ, PPARα also plays crucial roles in energy metabolism and responds to the circadian-clock system [9]. BMAL1 [51] and CLOCK [52] in the liver have been demonstrated to be upstream regulators of *PPARα* gene expression. PPARα also acts to regulate Rev-erb α in liver [53]. The *PPARα* gene has been identified as a circadian clock-controlled gene with a diurnal rhythmicity in the liver [54–56]. Therefore, we used the PPARα and PPARγ antagonists, GW6471 and GW9662, respectively to verify that the effects of Rosi on the circadian-clock system were through either the PPARα or PPARγ mechanisms. It was found that the PPARγ antagonist, GW9662, reversed the effects of Rosi on the circadian-clock system as well as the blood glucose response affected by IR. In contrast, the PPARα antagonist, GW6471, had a minor to no effect on the beneficial effect of Rosi on the circadian-clock system or blood glucose responses. A similar finding was observed in regards to the neuroprotective effects of Rosi on Huntington’s disease which showed that PPARγ, but not PPARα, plays an important role on rescuing motor dysfunction [57].

The present study also highlighted the interplay between PPARγ, the stability of the circadian-clock system, and energy metabolism. Rosi was administered in the drinking water from 20- to 60-weeks old and this chronic Rosi treatment was able to attenuate or delay the IR response in the intraperitoneal glucose and insulin tolerance tests as well as changes in the circadian-clock system. This confirms that PPARγ agonists are beneficial for IR and also for the circadian-clock system [21]. Furthermore, DM status (60-weeks old) disrupted and/or elevated the rhythmic patterns of the circadian-clock system and downstream genes in the liver. The chronic treatment with Rosi acted to stabilize the rhythmicity of the hepatic circadian-clock system to some degree.

Previous studies found that the hepatic circadian-clock system was impaired or dysfunctional in type 1 DM conditions [38–40]. The present study also revealed similar findings at 60-weeks old with long-term DM conditions. Notably, Zhang et al. found that over-expression of *Cry1* in the liver improved insulin sensitivity by lowering blood glucose levels in IR conditions of *db/db* mice [8]. In this study, the elevation of *Cry1* mRNA levels was found in IR conditions at 32- and 48-weeks old and in DM conditions at 60-weeks old. This study suggests that the elevated gene expression in the hepatic circadian-clock system in the IR condition may act to rescue or compensate for glucose imbalance and improve the IR and/or DM condition. However, gene expression changes for rescuing events will be lost in severe or untreated DM conditions. This study further confirmed that enhancing or maintaining the stability of the circadian-clock system in advance might provide therapeutic benefit to individuals with IR and/or DM. The other question in this study is how gene expression levels of the circadian-clock system were elevated without significant changes in the phase. Determining whether the sampling time points were insufficient is required to approach this problem in a future study.

The hypothalamic suprachiasmatic nuclei enable control of peripheral oscillators through neuronal and humoral signals. It also exerts control by driving the rest-activity and feeding-fasting rhythms to regulate the liver’s physiological functions. However, energy imbalances (high-fat diet induced obesity) or restricted feeding time (daytime feeding in rodents) are able to influence the locomotor rhythmicity but have only minor effects on the rhythmic activity or gene expression in the suprachiasmatic nuclei [33, 58]. Moreover, feeding or food intake is a powerful cue to drive the phase of hepatic gene expression [58, 59]. Insulin might be involved in the regulation of gene expression related to feeding patterns. Recently, a few studies have shown that insulin acts as a molecular synchronizer for the hepatic circadian-clock systems and is associated with feeding behavior [58–60]. The present study is in agreement with this point of view and suggests that insulin sensitizers might have similar effects as insulin. Whether hepatic gene expression in the IR and/or DM condition was directly or indirectly related to or controlled by the suprachiasmatic nuclei is an interesting topic of future study. Malfunction of the suprachiasmatic nuclei or the absence of central clock activity leads to the development of IR [61]. A recent study showed a decrease in circadian sensitivity to light in the retina at low intensities during chronic DM status [41]. Whether the retinal damage contributed to the changes of the hepatic circadian-clock system in the IR and/or DM conditions is an interesting issue, but it is a distant interaction beyond the scope of this study.

In conclusion, these results indicate that the molecular and biochemical responses of energy metabolism and the hepatic circadian-clock system are strongly mediated by the PPARγ agonist, Rosi. The chronic and low dose administration of the PPARγ agonist is sufficient to attenuate and preserve the markedly reduced IR and/or DM-related changes in the hepatic circadian-clock system and maintain glucose homeostasis. Employing the insulin sensitizing PPARγ agonist Rosi effectively improved the physiological parameters in the IR and DM conditions. The PPARγ agonist stabilized the altered hepatic circadian-clock and related gene expression, implying that the peripheral circadian-clock system, especially in the liver, is an attractive therapeutic candidate for treating the metabolic syndrome.

## Supporting Information

S1 Fig Effect of postnatal day two-streptozotocin (PND2-STZ) treatment on body weight (A), fasting blood glucose (B) and serum insulin (C) levels in male mice from 3 to 48 weeks old. Values represent the mean ± SEM (71 animals from the control group (open symbol); 78 animals from the PND2-STZ group (filled)). * *p*<0.05 compared with mice injected with citrate buffer as the controls on the same day.
(TIF)

S2 Fig Blood glucose response to intraperitoneal glucose tolerance (A-E) and intraperitoneal insulin tolerance tests (F-J) in mice at 16 (A,F), 24 (B,G), 32 (C,H), 40 (D,I) and 48 (E,J) weeks with postnatal day 2-streptozotocin (PND2-STZ) treatment. Values are presented as the mean ± SEM (14~15 animals for each time point in the control group; 15~16 animals for each time point in the PND2-STZ group). * *p*<0.05 compared to mice injected with citrate buffer as the control at the same time points on the same day. Open circle: the control group; filled triangle: the PND2-STZ group.
(TIF)

S3 Fig Effect of rosiglitazone (Rosi) treatment with either PPARα antagonist (GW6471) or PPARγ antagonist (GW9662) for two weeks on the blood glucose response to intraperitoneal glucose tolerance (A-B, E-F) and intraperitoneal insulin tolerance (C-D, G-H) in normal 32 week old male mice (A-D) or 48 weeks old male mice (E-H). Values are presented as the mean ± SEM (4~6 animals for each time point in A, C, E and G panels; 35 animals for the control group and 22~36 animals for the control groups with further treatments in the B, D, F and H panels). Open circle: the control group treated for two weeks with saline; filled triangle: the control group treated for two weeks with Rosi; filled square: the control group treated for two weeks with Rosi and PPARα antagonist, GW6471; reversed open triangle: the control group treated for two weeks with Rosi and PPARγ antagonist, GW9662.
(TIF)

S4 Fig Expression levels of *period 1* (*Per1*), *Per2*, *brain and muscle Arnt-like protein-1* (*Bmal1*), *cryptochrome 1* (*Cry1*), and *casein kinase 1ε* (*CK1ε*) in the livers of mice at 32 (left panel) or 48 weeks of age (right panel) undergoing treatment with rosiglitazone (Rosi), Rosi with PPARα antagonist (GW6471) or Rosi with PPARγ antagonist (GW9662) for two weeks. Values are presented as the mean ± SEM (3~8 animals for each time point). Open circle: the control group treated for two weeks with saline; filled triangle: the control group treated for two weeks with Rosi; filled square: the control group treated for two weeks with Rosi and PPARα antagonist, GW6471; reversed open triangle: the control group treated for two weeks with Rosi and PPARγ antagonist, GW9662.
(TIF)

S1 Table Sequences of primers for qPCR. Bmal1; brain and muscle Arnt-like protein-1. CK1; casein kinase 1. Clock; circadian locomotor output cycles kaput. Cry; cryptochrome. DBP; albumin D-site-binding protein. E4BP4; E4 binding protein 4. Per; period. PEPCK; phosphoenolpyruvate carboxykinase. PDK4; pyruvate dehydrogenase kinase 4. PGC1; peroxisome proliferator-activated receptor-γ coactivator 1. PPAR; peroxisome proliferator-activated receptor. Rev-erb; nuclear receptor subfamily 1, group D.
(PDF)

S2 Table Circadian characteristics of the cosine-fitted profiles of gene expression for different ages and treatments of mice, as depicted in Figs. 2–3 and 6. Mesor refers to the midline estimating statistic of rhythm, a rhythm-adjusted mean; amplitude (A) refers to a measure of the extent of predictable change within one cycle; and acrophase (Φ) refers to a measure of the timing of overall high values recurring in each cycle. The *P* value refers to the probability from the zero-amplitude (no rhythm) test. hh:mm; hour:minutes. wk; weeks. Cont; control. DMSO; dimethyl sulfoxide. IR; insulin resistance. GW6471; PPARα antagonist. GW9662; PPARγ antagonist. Rosi; rosiglitazone treatment. *Bmal1*; *brain and muscle Arnt-like protein-1*. *CK1*; *casein kinase 1*. *Clock*; *circadian locomotor output cycles kaput*. *Cry*; *cryptochrome*. *DBP*; *albumin D-site-binding protein*. *E4BP4*; *E4 binding protein 4*. *Per*; *period*. *PEPCK*; *phosphoenolpyruvate carboxykinase*. *PDK4*; *pyruvate dehydrogenase kinase 4*. *PGC1*; *peroxisome proliferator-activated receptor-γ coactivator 1*. *PPAR*; *peroxisome proliferator-activated receptor*. *Rev-erb*; *nuclear receptor subfamily 1*, *group D*.
(PDF)
