# Supplementary figures and images for: Hepatic Circadian-Clock System Altered by Insulin Resistance, Diabetes and Insulin Sensitizer in Mice
Source: PLoS One. 2015 Mar 23;10(3):e0120380. doi: 10.1371/journal.pone.0120380 (PMC4370469; doi:10.1371/journal.pone.0120380)

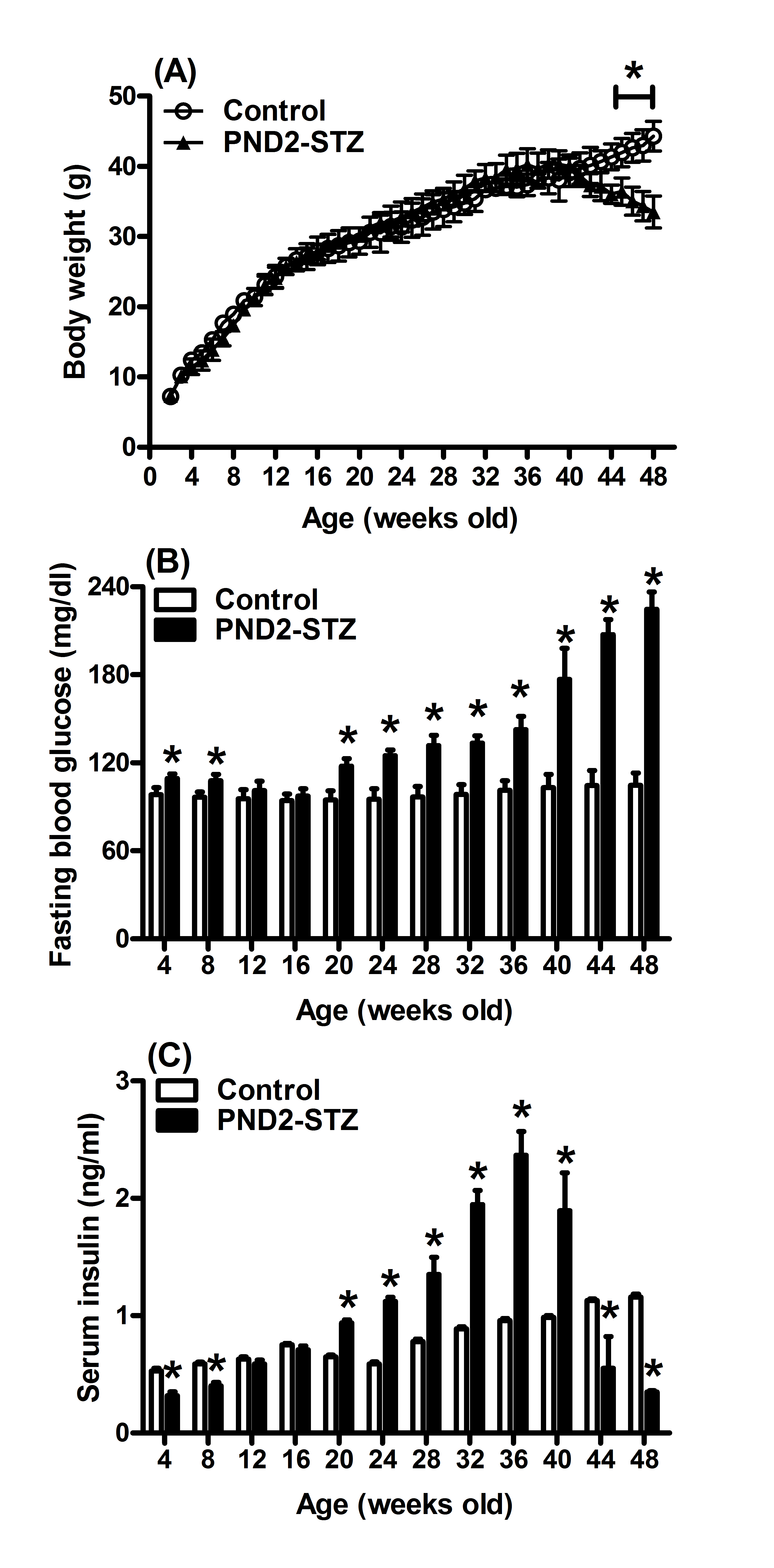

Supplement: S1 Fig — Values represent the mean ± SEM (71 animals from the control group (open symbol); 78 animals from the PND2-STZ group (filled)). * p<0.05 compared with mice injected with citrate buffer as the controls on the same day. (TIF) [file pone.0120380.s001.tif]

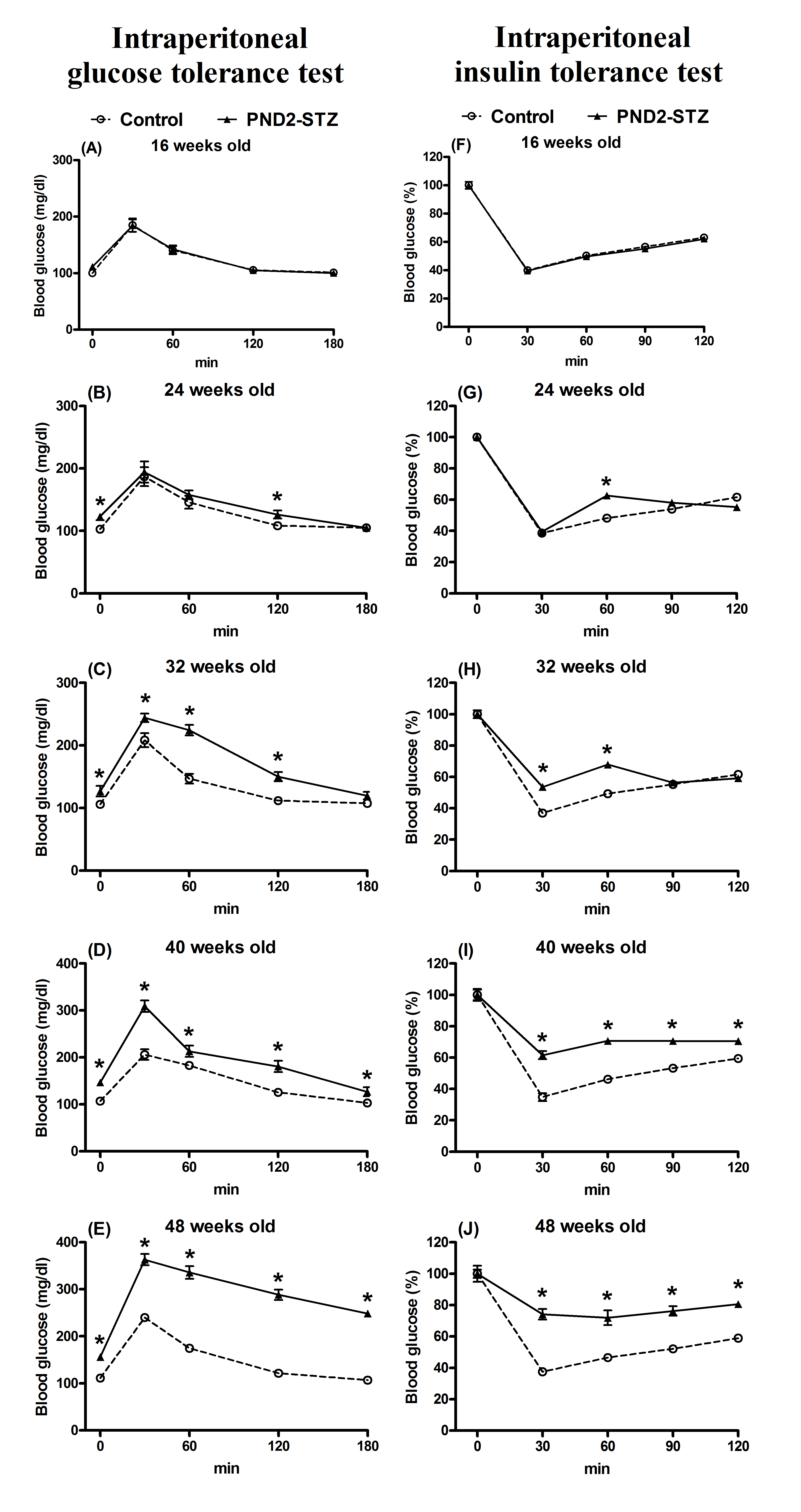

Supplement: S2 Fig — Values are presented as the mean ± SEM (14~15 animals for each time point in the control group; 15~16 animals for each time point in the PND2-STZ group). * p<0.05 compared to mice injected with citrate buffer as the control at the same time points on the same day. Open circle: the control group; filled triangle: the PND2-STZ group. (TIF) [file pone.0120380.s002.tif]

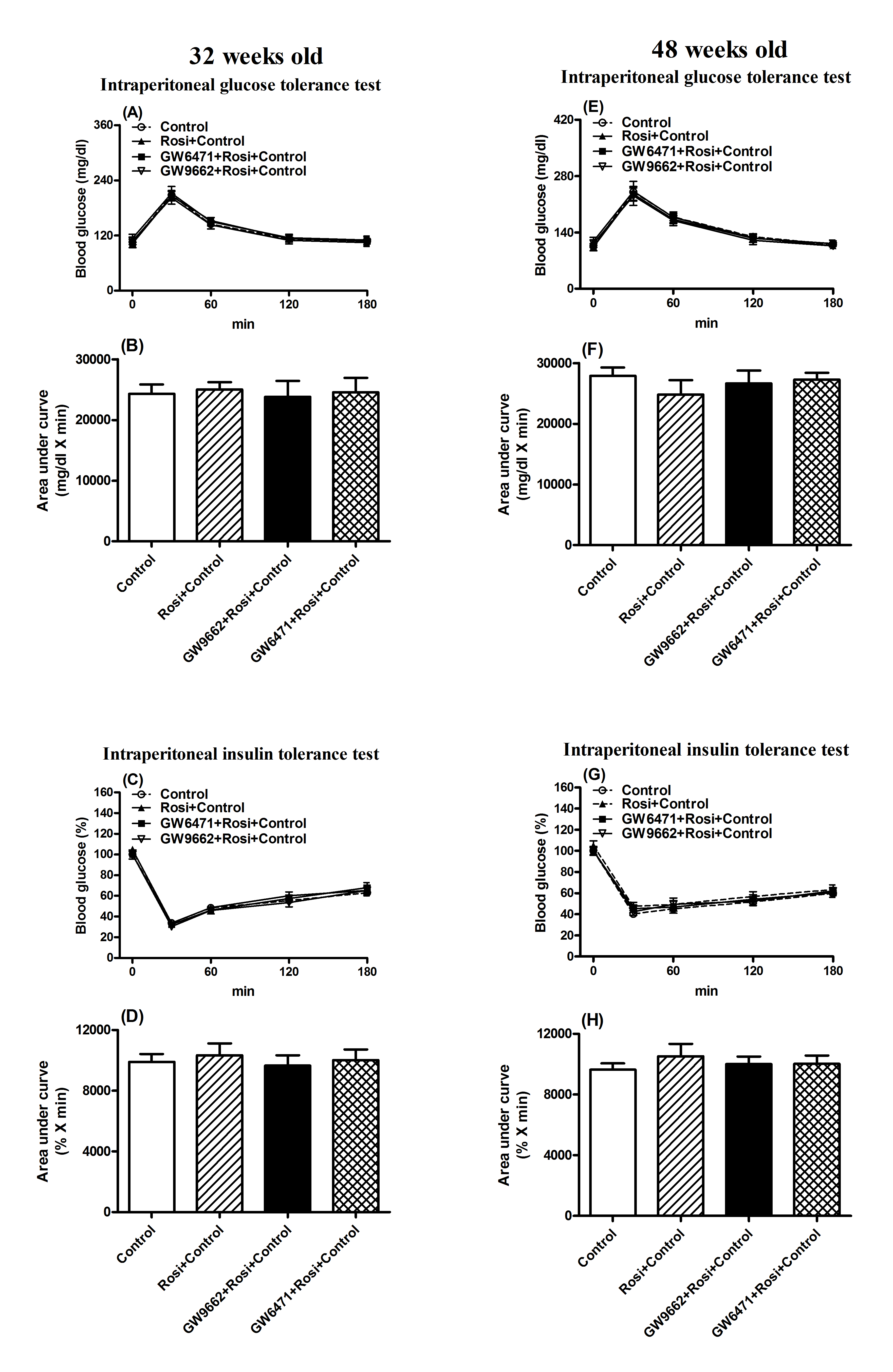

Supplement: S3 Fig — Values are presented as the mean ± SEM (4~6 animals for each time point in A, C, E and G panels; 35 animals for the control group and 22~36 animals for the control groups with further treatments in the B, D, F and H panels). Open circle: the control group treated for two weeks with saline; filled triangle: the control group treated for two weeks with Rosi; filled square: the control group treated for two weeks with Rosi and PPARα antagonist, GW6471; reversed open triangle: the control group treated for two weeks with Rosi and PPARγ antagonist, GW9662. (TIF) [file pone.0120380.s003.tif]

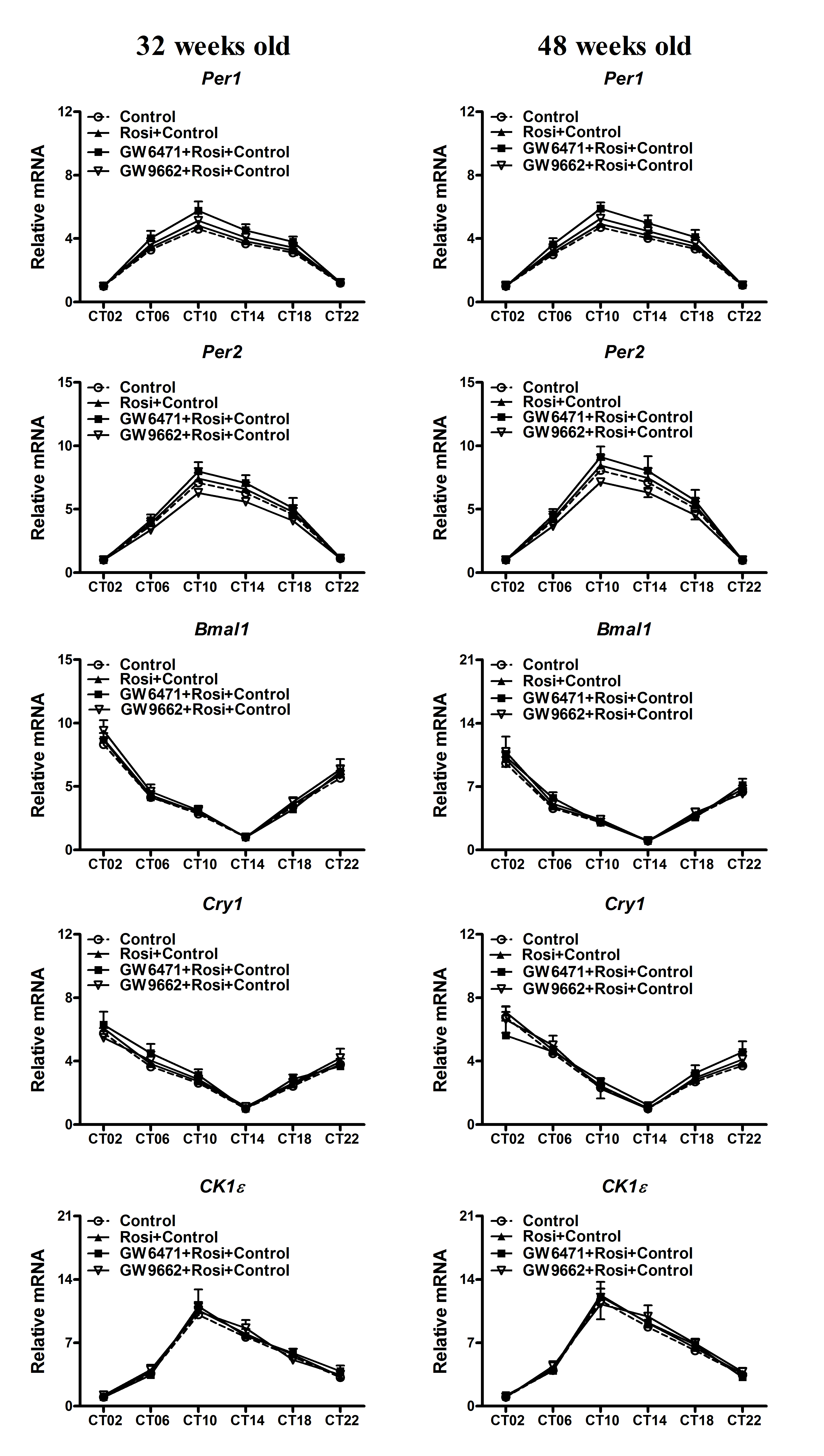

Supplement: S4 Fig — Values are presented as the mean ± SEM (3~8 animals for each time point). Open circle: the control group treated for two weeks with saline; filled triangle: the control group treated for two weeks with Rosi; filled square: the control group treated for two weeks with Rosi and PPARα antagonist, GW6471; reversed open triangle: the control group treated for two weeks with Rosi and PPARγ antagonist, GW9662. (TIF) [file pone.0120380.s004.tif]
