# Supplementary material for: Hepatic Circadian-Clock System Altered by Insulin Resistance, Diabetes and Insulin Sensitizer in Mice
Source: PLoS One. 2015 Mar 23;10(3):e0120380. doi: 10.1371/journal.pone.0120380 (PMC4370469; doi:10.1371/journal.pone.0120380)
Supplement: S1 Table — Bmal1; brain and muscle Arnt-like protein-1. CK1; casein kinase 1. Clock; circadian locomotor output cycles kaput. Cry; cryptochrome. DBP; albumin D-site-binding protein. E4BP4; E4 binding protein 4. Per; period. PEPCK; phosphoenolpyruvate carboxykinase. PDK4; pyruvate dehydrogenase kinase 4. PGC1; peroxisome proliferator-activated receptor-γ coactivator 1. PPAR; peroxisome proliferator-activated receptor. Rev-erb; nuclear receptor subfamily 1, group D. (PDF) [file pone.0120380.s005.pdf]

S1 Table. Sequences of primers for qPCR

Gene	Primer sequence
β-actin	5'- GCTGAGAGGGAAATCGT -3'
	5'- CGTCAGGCAGCTCATAG -3'
Per1	5'- GTGGGCTTGACACCTCTTCT -3''
	5'- TGCTTTAGATCGGCAGTGGT -3'
Per2	5'- GTTCCAGGCTGTGGATGAA -3'
	5'- GGCGTCTCGATCAGATCCT -3'
Per3	5'- GTCTGGAAGGTTAGTGCACATTTCT -3'
	5'- CACACTTGCCTCCGAAATAACTC -3'
Bmal1	5'- ATTCCAGGGGGAACCAGA -3'
	5'- GAAGGTGATGACCCTCTTATCCT -3'
Clock	5'- CACAGGCCAGCACATGAT -3'
	5'- CACTCATTACACTCTGTTGACTCTGA -3'
Cry1	5'- ATCGTGCGCATTTCACATAC -3'
	5'- TCCGCCATTGAGTTCTATGAT -3'
Cry2	5'- GGGAGCATCAGCAACACAG -3'
	5'- GCTTCCAGCTTGCGTTTG -3'
PEPCK	5'- CATATGCTGATCCTGGGCATAAC -3'
	5'- CAAACTTCATCCAGGCAATGTC -3'
PDK4	5'- GCGATGTGGTAGCAGTAGTC -3'
	5'- ATGTGGTGAAGGTGTGAAGG -3'
PGC1α	5'- AGCCGTGACCACTGACAACGAG -3'
	5'- GCTGCATGGTTCTGAGTGCTAAG -3'
PGC1β	5'- CGCTCCAGGAGACTGAATCCAG -3'
	5'- CTTGACTACTGTCTGTGAGGC -3'
E4BP4	5'- TCACAAAGAACTGAGCAGCAAAAC -3'
	5'- AACCTTATAGCCACCGTCTTTGAC -3'
DBP	5'- ACAGCAAGCCCAAAGAACC -3'
	5'- GAGGGCAGAGTTGCCTTG -3'
CK1ε	5'- GCCTCTATCAACACCCACCT -3'
	5'- GGAGCCCAGGTTGAAGTACA -3'
Rev-erb α	5'- CTACTGGCTCCCTCACCCAGGA -3'
	5'- GACACTCGGCTGCTGTCTTCCA -3'
Rev-erb β	5'- CGCACATTGCCGATATAGGAGG -3'
	5'- GAGACTGCCACCACCACGTACT -3'
PPARα	5'- GGGTACCACTACGGAGTTCACG -3'

	5'- CAGACAGGCACTTGTGAAAACG -3'
PPARγ	5'- TTAGATGACAGTGACTTGGC -3'
	5'- TCTTCTGGAGCACCTTGG -3'

Bmal1; *brain and muscle Arnt-like protein-1. CK1*; *casein kinase 1. Clock*; *circadian locomotor output cycles kaput. Cry*; *cryptochrome. DBP*; *albumin D-site-binding protein. E4BP4*; *E4 binding protein 4. Per*; *period. PEPCK*; *phosphoenolpyruvate carboxykinase. PDK4*; *pyruvate dehydrogenase kinase 4. PGC1*; *peroxisome proliferator-activated receptor-γ coactivator 1. PPAR*; *peroxisome proliferator-activated receptor. Rev-erb*; *nuclear receptor subfamily 1, group D.*
