# Supplementary material for: Hepatic Circadian-Clock System Altered by Insulin Resistance, Diabetes and Insulin Sensitizer in Mice
Source: PLoS One. 2015 Mar 23;10(3):e0120380. doi: 10.1371/journal.pone.0120380 (PMC4370469; doi:10.1371/journal.pone.0120380)
Supplement: S2 Table — Mesor refers to the midline estimating statistic of rhythm, a rhythm-adjusted mean; amplitude (A) refers to a measure of the extent of predictable change within one cycle; and acrophase (Φ) refers to a measure of the timing of overall high values recurring in each cycle. The P value refers to the probability from the zero-amplitude (no rhythm) test. hh:mm; hour:minutes. wk; weeks. Cont; control. DMSO; dimethyl sulfoxide. IR; insulin resistance. GW6471; PPARα antagonist. GW9662; PPARγ antagonist. Rosi; rosiglitazone treatment. Bmal1; brain and muscle Arnt-like protein-1. CK1; casein kinase 1. Clock; circadian locomotor output cycles kaput. Cry; cryptochrome. DBP; albumin D-site-binding protein. E4BP4; E4 binding protein 4. Per; period. PEPCK; phosphoenolpyruvate carboxykinase. PDK4; pyruvate dehydrogenase kinase 4. PGC1; peroxisome proliferator-activated receptor-γ coactivator 1. PPAR; peroxisome proliferator-activated receptor. Rev-erb; nuclear receptor subfamily 1, group D. (PDF) [file pone.0120380.s006.pdf]

S2 Table. Circadian characteristics of the cosine-fitted profiles of gene expression for different ages and treatments of mice, as depicted in Figs 2-3 and 6

Gene	Age	Group	Mesor	Amplitude, A	Acrophase, Φ (hh:mm)	Percentage of rhythm	*P*-value
Per1	32 wk	Cont	2.8125	1.7641	11:36	83.6	0.0227
	32 wk	IR	5.7348	4.3512	12:01	85.1	0.0196
	32 wk	IR + Rosi	3.6083	2.8433	11:53	96.5	0.0021
	32 wk	IR + GW9662/Rosi	7.0221	5.4274	12:05	81.8	0.0269
	32 wk	IR + GW6471/Rosi	3.6934	2.9765	11:40	90.7	0.0095
	48 wk	Cont	2.8551	1.9259	12:01	89.8	0.0109
	48 wk	IR	6.9455	4.0209	11:54	75.3	0.0431
	48 wk	IR + Rosi	5.3634	4.6215	11:59	85.3	0.0192
	48 wk	IR + GW9662/Rosi	7.9919	4.6718	11:50	76.7	0.0393
	48 wk	IR + GW6471/Rosi	8.2091	4.9222	12:02	87.8	0.0143
	60 wk	Cont (48 wk)	2.8551	1.9259	12:01	89.8	0.0109
	60 wk	IR + DMSO	-	-	-	2.2	0.3418
	60 wk	IR + Rosi (40 wk)	3.6714	3.1647	11:41	81.9	0.0266
Per2	32 wk	Cont	3.5817	2.8251	12:12	95.7	0.0029
	32 wk	IR	5.8191	3.9562	12:38	81.7	0.0271
	32 wk	IR + Rosi	3.3766	2.6923	12:06	95.9	0.0027
	32 wk	IR + GW9662/Rosi	6.5848	4.7079	12:38	81.7	0.0271
	32 wk	IR + GW6471/Rosi	3.7115	3.1455	12:04	95.7	0.0030
	48 wk	Cont	3.9473	3.3174	12:11	95.6	0.0030
	48 wk	IR	6.9181	4.3634	12:15	85.5	0.0188

	48 wk	IR + Rosi	6.3448	4.1106	12:33	84.4	0.0211
	48 wk	IR + GW9662/Rosi	8.0092	4.9844	12:10	87.2	0.0155
	48 wk	IR + GW6471/Rosi	6.7104	3.8282	13:02	86.5	0.0168
	60 wk	Cont (48 wk)	3.9473	3.3174	12:11	95.6	0.0030
	60 wk	IR + DMSO	-	-	-	0.1	0.5057
	60 wk	IR + Rosi (40 wk)	4.9209	4.3558	11:34	87.9	0.0142
Per3	32 wk	Cont	3.4194	3.107	12:54	81.1	0.0284
	32 wk	IR	3.5247	3.0679	12:42	89.0	0.0123
	32 wk	IR + Rosi	3.2268	2.9364	12:51	78.9	0.0337
	32 wk	IR + GW9662/Rosi	3.9044	3.5567	12:40	87.0	0.0159
	32 wk	IR + GW6471/Rosi	3.3665	3.1274	12:48	87.7	0.0147
	48 wk	Cont	4.3062	4.2325	12:40	87.0	0.0159
	48 wk	IR	5.9995	5.2405	12:56	91.5	0.0083
	48 wk	IR + Rosi	4.7844	5.0366	12:40	87.1	0.0171
	48 wk	IR + GW9662/Rosi	6.9661	6.1641	12:44	91.4	0.0083
	48 wk	IR + GW6471/Rosi	4.7258	4.6331	12:25	88.4	0.0133
Bmal1	32 wk	Cont	4.2321	3.0893	01:15	80.2	0.0305
	32 wk	IR	7.4467	3.1832	01:50	90.0	0.0105
	32 wk	IR + Rosi	4.0590	2.5382	01:26	83.2	0.0237
	32 wk	IR + GW9662/Rosi	8.1882	3.8070	02:25	85.3	0.0193
	32 wk	IR + GW6471/Rosi	4.3236	3.2385	01:07	92.6	0.0067

	48 wk	Cont	4.7212	3.6272	01:14	80.0	0.0311
	48 wk	IR	8.5216	3.7880	01:50	90.0	0.0105
	48 wk	IR + Rosi	6.9902	3.7215	02:44	93.2	0.0058
	48 wk	IR + GW9662/Rosi	9.8007	4.5077	01:51	90.1	0.0101
	48 wk	IR + GW6471/Rosi	7.9784	4.4285	02:40	93.0	0.0063
	60 wk	Cont (48 wk)	4.7212	3.6272	01:14	80.0	0.0311
	60 wk	IR + DMSO	-	-	-	-	0.5476
	60 wk	IR + Rosi (40 wk)	-	-	-	59.2	0.0891
Clock	32 wk	Cont	-	-	-	-	0.5205
	32 wk	IR	-	-	-	-	0.8654
	32 wk	IR + Rosi	-	-	-	-	0.7306
	32 wk	IR + GW9662/Rosi	-	-	-	-	0.5611
	32 wk	IR + GW6471/Rosi	-	-	-	-	0.5388
	48 wk	Cont	-	-	-	-	0.7769
	48 wk	IR	-	-	-	-	0.8878
	48 wk	IR + Rosi	-	-	-	-	0.7050
	48 wk	IR + GW9662/Rosi	-	-	-	9.7	0.3035
	48 wk	IR + GW6471/Rosi	-	-	-	55.6	0.1048
Cry1	32 wk	Cont	3.2084	1.9912	02:02	83.9	0.0222
	32 wk	IR	6.0577	2.6153	01:17	83.2	0.0236
	32 wk	IR + Rosi	2.7818	1.8313	02:12	86.5	0.0168

	32 wk	IR + GW9662/Rosi	6.8686	3.1122	01:17	83.2	0.0236
	32 wk	IR + GW6471/Rosi	2.9371	1.8597	01:56	91.7	0.0079
	48 wk	Cont	3.5000	2.4529	02:11	81.0	0.0287
	48 wk	IR	7.8337	3.7036	01:17	83.2	0.0236
	48 wk	IR + Rosi	5.4884	3.7215	02:16	89.3	0.0116
	48 wk	IR + GW9662/Rosi	8.9821	4.4073	01:17	83.1	0.0251
	48 wk	IR + GW6471/Rosi	5.6912	3.5849	02:01	92.2	0.0072
	60 wk	Cont (48 wk)	3.5000	2.4529	02:11	81.0	0.0287
	60 wk	IR + DMSO	8.3547	3.0596	01:21	83.8	0.0223
	60 wk	IR + Rosi (40 wk)	4.7544	3.3114	03:31	84.1	0.0217
Cry2	32 wk	Cont	2.7683	2.1277	12:04	66.8	0.0230
	32 wk	IR	2.6794	2.0527	11:55	59.6	0.0489
	32 wk	IR + Rosi	2.9468	2.3840	12:20	62.7	0.0358
	32 wk	IR + GW9662/Rosi	2.8355	2.4403	12:11	62.0	0.0405
	32 wk	IR + GW6471/Rosi	2.7974	2.4976	12:13	73.6	0.0099
	48 wk	Cont	3.6062	3.1138	12:31	75.3	0.0080
	48 wk	IR	3.7063	3.4874	12:37	80.0	0.0040
	48 wk	IR + Rosi	3.3669	3.2284	12:25	75.2	0.0081
	48 wk	IR + GW9662/Rosi	3.7372	3.5706	12:43	80.4	0.0038
	48 wk	IR + GW6471/Rosi	3.5332	3.5164	12:37	79.4	0.0044
PEPCK	32 wk	Cont	3.8091	2.8239	13:05	95.7	0.0030

	32 wk	IR	7.2434	3.5679	13:22	92.2	0.0072
	32 wk	IR + Rosi	4.1621	3.0078	13:06	94.1	0.0047
	32 wk	IR + GW9662/Rosi	8.2796	4.2458	13:22	92.2	0.0072
	32 wk	IR + GW6471/Rosi	4.0962	2.9599	12:52	86.0	0.0179
	48 wk	Cont	4.2178	3.3119	13:04	95.6	0.0030
	48 wk	IR	8.2796	4.2458	13:22	92.1	0.0075
	48 wk	IR + Rosi	6.2795	4.3818	12:44	87.9	0.0143
	48 wk	IR + GW9662/Rosi	9.5127	5.0525	13:22	92.0	0.0079
	48 wk	IR + GW6471/Rosi	6.4659	4.2806	12:27	84.7	0.0205
	60 wk	Cont (48 wk)	4.2178	3.3119	13:04	95.6	0.0030
	60 wk	IR + DMSO	-	-	-	-	0.6200
	60 wk	IR + Rosi (40 wk)	-	-	-	58.6	0.0798
PDK4	32 wk	Cont	3.4088	2.2186	01:15	83.7	0.0225
	32 wk	IR	8.4648	3.2748	01:33	91.3	0.0085
	32 wk	IR + Rosi	7.8316	3.7408	01:36	90.1	0.0104
	32 wk	IR + GW9662/Rosi	8.6164	3.2002	01:21	75.8	0.0417
	32 wk	IR + GW6471/Rosi	8.3847	3.4371	01:51	97.8	0.0011
	48 wk	Cont	3.7414	2.5912	01:14	83.8	0.0224
	48 wk	IR	9.7331	3.8970	01:33	91.3	0.0085
	48 wk	IR + Rosi	9.8130	4.1203	01:34	95.5	0.0032
	48 wk	IR + GW9662/Rosi	10.2424	3.9762	01:28	90.6	0.0096
	48 wk	IR + GW6471/Rosi	10.3375	3.9115	01:27	85.6	0.0186

PGC1α	32 wk	Cont	4.5514	3.5423	14:00	93.3	0.0057
	32 wk	IR	12.8008	6.2404	14:22	93.5	0.0055
	32 wk	IR + Rosi	4.7852	3.6973	13:56	94.6	0.0041
	32 wk	IR + GW9662/Rosi	14.5597	6.9049	14:14	91.5	0.0081
	32 wk	IR + GW6471/Rosi	4.6879	3.3999	13:55	94.0	0.0048
	48 wk	Cont	5.7554	4.9067	14:01	92.9	0.0062
	48 wk	IR	17.3827	8.837	14:24	93.4	0.0059
	48 wk	IR + Rosi	10.6984	7.1321	13:29	99.2	0.0004
	48 wk	IR + GW9662/Rosi	20.3454	10.5160	14:23	93.3	0.0057
	48 wk	IR + GW6471/Rosi	11.0578	7.1680	13:24	93.4	0.0055
	60 wk	Cont (48 wk)	5.7554	4.9067	14:01	92.9	0.0062
	60 wk	IR + DMSO	-	-	-	-	0.6095
	60 wk	IR + Rosi (40 wk)	10.2935	7.0429	12:43	82.1	0.0261
PGC1β	32 wk	Cont	5.4274	3.9943	14:31	90.8	0.0093
	32 wk	IR	10.0309	4.4914	12:55	85.8	0.0182
	32 wk	IR + Rosi	5.5514	4.2120	14:32	88.6	0.0129
	32 wk	IR + GW9662/Rosi	11.5968	5.3447	12:57	86.0	0.0191
	32 wk	IR + GW6471/Rosi	5.5995	4.0244	14:40	84.0	0.0220
	48 wk	Cont	6.1436	4.7037	14:31	91.0	0.0089
	48 wk	IR	13.4602	6.3602	12:56	85.9	0.0187
	48 wk	IR + Rosi	10.4500	5.7670	13:00	79.5	0.0322

	48 wk	IR + GW9662/Rosi	15.6776	7.5687	12:55	85.8	0.0182
	48 wk	IR + GW6471/Rosi	11.7622	6.6317	13:09	86.0	0.0177
	60 wk	Cont (48 wk)	6.1436	4.7037	14:31	91.0	0.0089
	60 wk	IR + DMSO	-	-	-	-	0.6798
	60 wk	IR + Rosi (40 wk)	8.5136	4.1361	12:30	91.0	0.0090
E4BP4	32 wk	Cont	3.1578	2.2984	01:03	98.3	0.0008
	32 wk	IR	7.9851	2.9696	01:37	88.1	0.0138
	32 wk	IR + Rosi	3.0757	2.1574	01:16	94.8	0.0039
	32 wk	IR + GW9662/Rosi	9.3290	3.3495	01:55	87.8	0.0144
	32 wk	IR + GW6471/Rosi	3.1034	2.1731	00:55	93.9	0.0049
	48 wk	Cont	3.7819	3.1488	01:01	97.4	0.0015
	48 wk	IR	10.5631	4.2053	01:37	88.1	0.0138
	48 wk	IR + Rosi	7.1109	3.3828	01:20	81.2	0.0282
	48 wk	IR + GW9662/Rosi	12.2301	5.0043	01:37	88.0	0.0141
	48 wk	IR + GW6471/Rosi	7.4553	3.3000	01:52	72.8	0.0498
DBP	32 wk	Cont	9.1095	8.0618	13:41	93.0	0.0061
	32 wk	IR	15.7683	10.3142	13:59	83.3	0.0234
	32 wk	IR + Rosi	9.6495	8.3048	13:34	90.4	0.0099
	32 wk	IR + GW9662/Rosi	18.0909	11.6073	13:59	84.6	0.0207
	32 wk	IR + GW6471/Rosi	9.3263	8.2886	13:53	93.8	0.0051
	48 wk	Cont	12.2102	11.3072	13:41	92.9	0.0062

	48 wk	IR	21.5848	14.606	13:59	83.3	0.0234
	48 wk	IR + Rosi	20.9201	14.8500	14:01	90.0	0.0106
	48 wk	IR + GW9662/Rosi	22.1793	13.8831	14:04	86.3	0.0172
	48 wk	IR + GW6471/Rosi	21.8882	14.8433	14:06	84.4	0.0210
CK1ε	32 wk	Cont	5.1552	3.954	12:33	73.0	0.0492
	32 wk	IR	12.0668	7.4966	11:56	73.2	0.0489
	32 wk	IR + Rosi	5.1618	4.0381	12:27	79.5	0.0323
	32 wk	IR + GW9662/Rosi	12.1861	7.2046	11:44	73.1	0.0491
	32 wk	IR + GW6471/Rosi	5.4692	4.2063	12:32	77.8	0.0365
	48 wk	Cont	5.8196	4.6589	12:32	73.0	0.0495
	48 wk	IR	14.0194	8.9209	11:55	73.1	0.0491
	48 wk	IR + Rosi	10.9692	7.1229	11:35	73.4	0.0485
	48 wk	IR + GW9662/Rosi	15.1765	9.7481	11:59	79.8	0.0315
	48 wk	IR + GW6471/Rosi	11.4633	7.5072	11:24	73.3	0.0487
	60 wk	Cont (48 wk)	5.8196	4.6589	12:32	73.0	0.0495
	60 wk	IR + DMSO	-	-	-	-	0.5985
	60 wk	IR + Rosi (40 wk)	9.0386	7.2172	11:09	85.1	0.0197
Rev-erbα	32 wk	Cont	6.3671	4.9451	14:06	96.4	0.0023
	32 wk	IR	19.9164	11.8616	14:56	85.4	0.0190
	32 wk	IR + Rosi	6.4399	4.627	14:17	94.7	0.0040
	32 wk	IR + GW9662/Rosi	23.3605	14.1153	14:55	85.3	0.0188

	32 wk	IR + GW6471/Rosi	6.9901	5.8260	13:54	84.5	0.0208
	48 wk	Cont	7.2619	5.8347	14:06	96.6	0.0021
	48 wk	IR	23.3605	14.1153	14:53	85.2	0.0196
	48 wk	IR + Rosi	14.9901	8.9624	14:40	85.4	0.0189
	48 wk	IR + GW9662/Rosi	26.2923	15.0411	14:54	78.6	0.0344
	48 wk	IR + GW6471/Rosi	15.8316	9.3873	14:25	85.5	0.0188
	60 wk	Cont (48 wk)	7.2619	5.8347	14:06	96.6	0.0021
	60 wk	IR + DMSO	-	-	-	-	0.4187
	60 wk	IR + Rosi (40 wk)	14.7678	10.7521	14:23	97.9	0.0011
Rev-erbβ	32 wk	Cont	4.0612	3.7309	12:32	89.5	0.0113
	32 wk	IR	8.8715	4.7921	12:18	88.7	0.0127
	32 wk	IR + Rosi	4.3696	4.1056	12:11	88.7	0.0127
	32 wk	IR + GW9662/Rosi	10.2171	5.7026	12:18	88.7	0.0127
	32 wk	IR + GW6471/Rosi	4.2098	4.0039	12:22	94.5	0.0043
	48 wk	Cont	4.5178	4.3935	12:31	88.8	0.0125
	48 wk	IR	10.2171	5.7026	12:15	88.6	0.0129
	48 wk	IR + Rosi	8.6931	5.2786	12:47	88.6	0.0129
	48 wk	IR + GW9662/Rosi	11.6517	6.4869	12:13	90.0	0.0106
	48 wk	IR + GW6471/Rosi	10.5048	5.9130	12:30	87.7	0.0145
	60 wk	Cont (48 wk)	4.5178	4.3935	12:31	88.8	0.0125
	60 wk	IR + DMSO	-	-	-	-	0.3622
	60 wk	IR + Rosi (40 wk)	7.1409	6.6637	11:32	86.4	0.0171

PPARα	32 wk	Cont	5.6753	4.4607	12:32	82.0	0.0289
	32 wk	IR	12.4814	7.6705	12:30	89.2	0.0175
	32 wk	IR + Rosi	5.6788	4.5631	12:27	86.3	0.0193
	32 wk	IR + GW9662/Rosi	13.122	8.5354	12:23	95.3	0.0041
	32 wk	IR + GW6471/Rosi	6.6013	5.1383	12:54	90.7	0.0152
	48 wk	Cont	11.8142	9.1109	13:13	73.6	0.0386
	48 wk	IR	23.2368	16.3196	12:42	77.5	0.0308
	48 wk	IR + Rosi	16.6524	12.0519	13:04	71.9	0.0483
	48 wk	IR + GW9662/Rosi	24.4370	16.6206	12:37	77.2	0.0326
	48 wk	IR + GW6471/Rosi	18.6632	13.6186	13:04	71.8	0.0486
	60 wk	Cont (48 wk)	11.8142	9.1109	13:13	73.6	0.0386
	60 wk	IR + DMSO	-	-	-	-	0.4774
	60 wk	IR + Rosi (40 wk)	15.3295	11.0298	12:10	74.7	0.0413
PPARγ	32 wk	Cont	6.6561	5.8091	14:04	92.4	0.0034
	32 wk	IR	10.8478	6.7285	14:39	87.8	0.0191
	32 wk	IR + Rosi	14.3200	9.1163	14:11	96.1	0.0010
	32 wk	IR + GW9662/Rosi	7.0548	5.5764	14:15	89.7	0.0106
	32 wk	IR + GW6471/Rosi	14.3999	8.8699	14:37	98.6	0.0003
	48 wk	Cont	10.2011	8.4397	14:18	96.7	0.0008
	48 wk	IR	13.9281	8.9598	14:06	96.3	0.0009
	48 wk	IR + Rosi	17.0430	10.2497	13:29	96.5	0.0008

48 wk	IR + GW9662/Rosi	10.9796	9.1531	14:07	94.7	0.0017
48 wk	IR + GW6471/Rosi	18.0881	11.5000	13:24	98.6	0.0003
60 wk	Cont (48 wk)	10.2011	8.4397	14:18	96.7	0.0008
60 wk	IR + DMSO	-	-	-	-	0.5970
60 wk	IR + Rosi (40 wk)	19.1121	11.3502	14:03	93.0	0.0029

Mesor refers to the midline estimating statistic of rhythm, a rhythm-adjusted mean; amplitude (A) refers to a measure of the extent of predictable change within one cycle; and acrophase (Φ) refers to a measure of the timing of overall high values recurring in each cycle. The *P* value refers to the probability from the zero-amplitude (no rhythm) test. hh:mm; hour:minutes. wk; weeks. Cont; control. DMSO; dimethyl sulfoxide. IR; insulin resistance. GW6471; PPARα antagonist. GW9662; PPARγ antagonist. Rosi; rosiglitazone treatment. *Bmal1*; *brain and muscle Arnt-like protein-1*. *CK1*; *casein kinase 1*. *Clock*; *circadian locomotor output cycles kaput*. *Cry*; *cryptochrome*. *DBP*; *albumin D-site-binding protein*. *E4BP4*; *E4 binding protein 4*. *Per*; *period*. *PEPCK*; *phosphoenolpyruvate carboxykinase*. *PDK4*; *pyruvate dehydrogenase kinase 4*. *PGC1*; *peroxisome proliferator-activated receptor-γ coactivator 1*. *PPAR*; *peroxisome proliferator-activated receptor*. *Rev-erb*; *nuclear receptor subfamily 1, group D*.
